# Monolithically integrated solid-state vertical organic electrochemical transistors switching between neuromorphic and logic functions

**DOI:** 10.1126/sciadv.adt5186

**Published:** 2025-03-14

**Authors:** Tianming Li, Zhe Qu, Jiansong Si, Yeji Lee, Vineeth Kumar Bandari, Oliver G. Schmidt

**Affiliations:** ^1^ Research Center for Materials, Architectures, and Integration of Nanomembranes (Main), TU Chemnitz, 09126, Chemnitz, Germany.; ^2^ Material Systems for Nanoelectronics, TU Chemnitz, 09107, Chemnitz, Germany, TU Chemnitz, 09126 Chemnitz, Germany.; ^3^ International Institute for Intelligent Nanorobots and Nanosystems (IIINN), Fudan University, Shanghai 200438, P. R. China.

## Abstract

Manipulating the ionic-electronic coupling in organic electrochemical transistors (OECTs) offers opportunities for interesting phenomena and advanced applications but has not been systematically exploited. Here, we develop monolithically integrated solid-state vertical OECTs to fully explore polyelectrolyte’s strengths, enabling the OECTs to switch between neuromorphic and logic functions. This transition capability is achieved by mastering the complex transport of large-size polycations within the channel through well-designed drain electrodes. Frame drains positioned atop the organic channel act as ion barriers, regulating the penetration and relaxation of polycations. This regulation allows our multilevel synaptic OECTs to transform from short-term depression (STD) to STD-based long-term memory, and eventually to long-term depression (LTD). Conversely, placing frame drains beneath the channel exposes the polyelectrolyte fully, hence yielding high-density logic OECTs, which have been successfully used to construct unipolar integrated circuits such as NOT, NAND, and NOR gates. These achievements represent a substantial advancement in manipulating polyelectrolyte-based ionic-electronic interactions, introducing more possibilities beyond small ion-based OECTs.

## Teaser

Polyelectrolyte-based monolithic OECTs enable multifunctionality by regulating the complex electronic-ionic interactions.

## INTRODUCTION

Organic electrochemical transistors (OECTs) have great potential in bioelectronics, neuroelectronics, wearable electronics, and digital electronics due to their remarkably low-power consumption, high transconductance, biocompatibility, and flexibility (*1*–*3*). These functions rely on a shared process: ions from the electrolyte are injected into the organic-mixed ionic-electronic conducting (OMIEC) channel through gate modulation, thereby altering the channel’s doping state and subsequently changing its conductivity (*4*). Governed by ion modulation efficiency, the drain current-gate voltage (*I*_D_ versus *V*_G_) transfer curves of OECTs generally exhibit two primary forms: (i) negligible or small *I-V* hysteresis, applicable to logic circuits (*5*), biosensing (*6*), and electrophysiological recording (*7*) and (ii) large hysteresis windows observed during forward and reverse sweeps of the gate voltage, which provides a promising platform for building artificial neuromorphic hardware (*8*, *9*). The hysteretic behavior is commonly attributed to restricted ion movement within the channel (*10*), and several strategies (such as altering the composition and/or morphology of organic channels) have been developed to regulate the dynamics of small ions (such as Na^+^) in lateral OECTs to meet specific requirements (note S1) (*11*–*13*).

In addition, emerging vertically stacked OECT configurations, with the OMIEC channel sandwiched between the source and drain, have greatly reduced the channel length and decreased the transistor footprint (*14*–*16*). However, the impermeable top drain electrode in the vertical traverse architecture serves as a solid barrier for vertical ion transport, so ions can only penetrate inside and spread out laterally from the uncovered organic channel region. This limitation is not a substantial obstacle for small ions in liquid electrolytes (*17*) but presents both challenges and opportunities when accommodating large ions and/or solid-state electrolytes. Now, polyelectrolytes containing charged repeating units have been widely used in solid-state batteries (*18*, *19*), having excellent processability and tunable interfacial compatibility. However, their potential in OECTs has been overlooked. Although relatively slow ion transport can be expected, polyelectrolytes may provide more possibilities for regulating the interaction between the OMIEC matrix and large ions due to their complex ion transport properties, particularly in top electrode–covered vertical architectures.

**Fig. 1.**
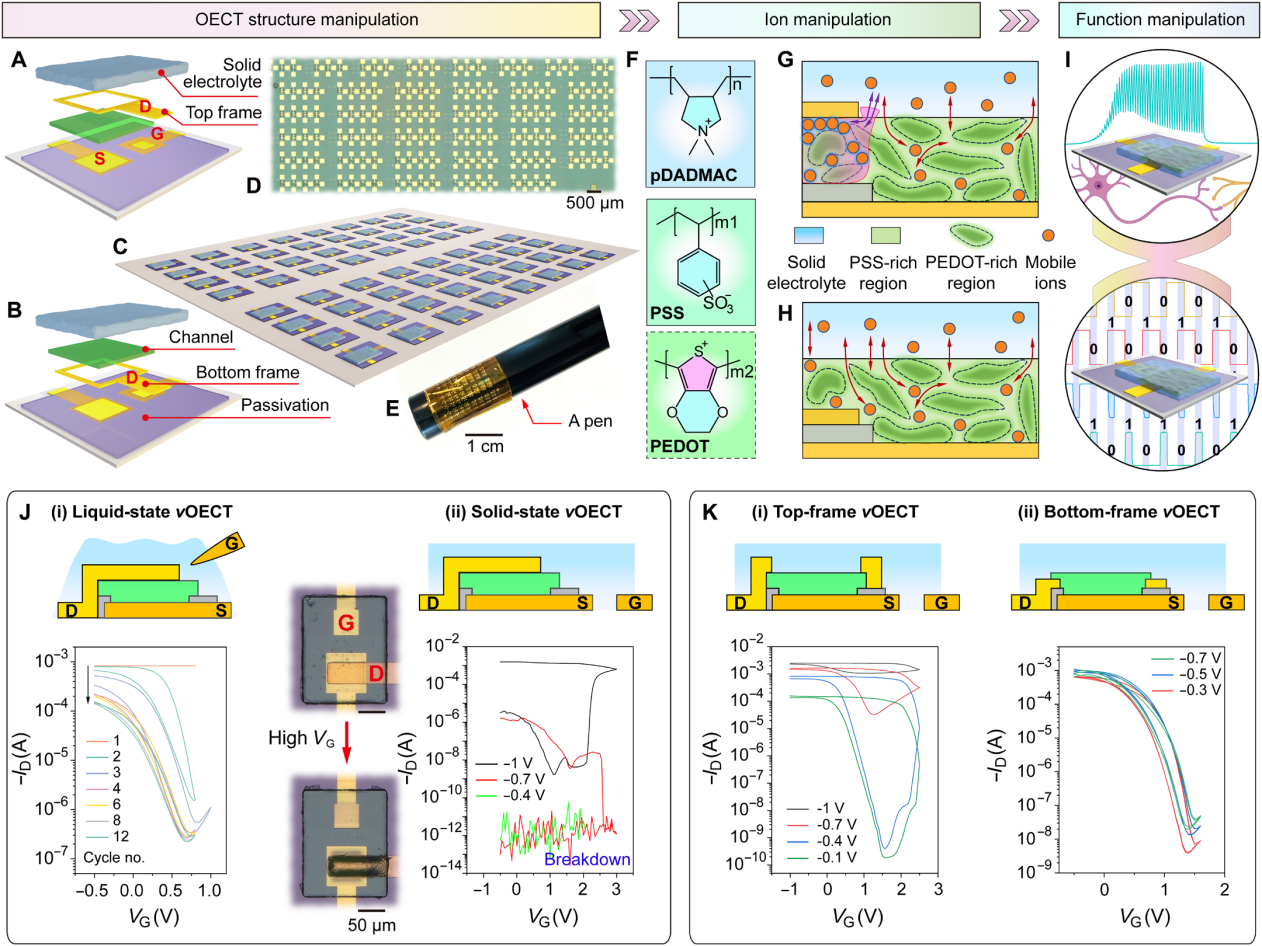
Structure manipulation, ion manipulation, and function manipulation of integrated solid-state vertical OECTs. (**A** and **B**) Schematics of structure manipulation of solid-state vertical OECTs based on top-frame and bottom-frame drains, respectively. D, S, and G represent drain, source, and gate electrodes, respectively. (**C**) Schematic of monolithic fabrication of solid-state vertical OECT array. (**D** and **E**) Optical images of solid-state OECT arrays on rigid and flexible substrates, respectively. (**F**) Chemical structures of the polyelectrolyte pDADMAC and p-type channel PEDOT:PSS. (**G** and **H**) Schematics of ion manipulation between channel and electrolyte through the drain shape and position. Blue, green, and gray regions represent electrolyte, channel, and passivation layer, respectively. The top drain acts as an ion transport barrier, whereas the bottom drain does not. (**I**) Schematics of function manipulation between neuromorphic and logic OECTs. (**J**) Schematics and performances of liquid-state (i) and solid-state (ii) vertical OECTs based on pDADMAC and PEDOT:PSS. The inset optical images show the state of solid-state vertical OECT before and after the damage caused by high gate voltage. (**K**) Schematics and performances of top-frame drain-based (i) and bottom-frame drain-based (ii) vertical OECTs. PEDOT:PSS thickness in (J) and (K) is ~30 nm.

Here, we report the development of monolithically integrated solid-state vertical OECTs on both rigid and flexible substrates, capable of switching between neuromorphic and logic functions by precisely manipulating the polycation transport within the OMIEC channel (Fig. 1). As a crucial initial step toward full integration, the cationic polyelectrolyte poly(diallyldimethylammonium chloride (pDADMAC) and p-type channel poly(3,4-ethylendioxythiophen):poly(styrolsulfonat) (PEDOT:PSS) are successfully patterned into microscale structures. The key to effectively manipulating the neuromorphic-logic functions lies in controlling the shape of the drain and its relative position to the channel, allowing for different ion transport modes of the large-size polycations. On the one hand, with a dense planar or frame drain positioned atop the PEDOT:PSS channel, the vertical solid-state OECTs behave as artificial synapses (Fig. 1, A, G, and I). By playing with the ion penetration and relaxation amplitudes, the synaptic OECTs exhibit plasticity transforming from short-term depression (STD) to STD-based long-term memory and eventually to long-term depression (LTD). On the other hand, when the frame drain is located beneath the PEDOT:PSS channel, the drain no longer acts as an ion blocker and can provide a partially vertical electric field across the channel (Fig. 1, B, H, and I). This enables the solid-state OECTs with relatively fast switching (several milliseconds), high transconductance (above 20 mS), large on/off ratio (six orders of magnitude), high integration density (>20,000 transistors cm^−2^) with a yield of 100%. On the basis of this technology, Morse code communication, Pavlovian associative learning, unipolar integrated circuits (including NOT, NAND and NOR gates), and electrophysiological signal recording are successfully demonstrated.

## RESULTS

### Design strategies for solid-state vertical OECTs

Initially, we dropped aqueous pDADMAC onto the vertical traverse Au (D)/PEDOT:PSS/Au (S) structure and found that several dedoping/redoping cycles were required to achieve relatively steady *I*_D_ versus *V*_G_ curves [Fig. 1J (i)]. This phenomenon is consistent with previous measurements (*20*) and our lateral OECTs based on pDADMAC solution (fig. S1), which has been attributed to hydration of the polymer film during the initial cycles. This observation indicates that (i) the polyelectrolyte pDADMAC is capable of gating PEDOT:PSS-based vertical OECTs, but (ii) the devices suffer from poor stability in the aqueous environment. When using small ion-based phosphate-buffered saline (PBS) as the liquid-state electrolyte for our vertical OECTs, only about one cycle was needed to reach reproducible curves (fig. S2). The slow stabilization with aqueous pDADMAC electrolyte suggests that pDADMAC can spread into the PEDOT:PSS film but at a slower rate compared to PBS.

Solid-state electrolytes have been reported to improve the operational stability of OECTs (*21*, *22*). Therefore, we spent efforts on patterning pDADMAC (see Materials and Methods) to construct solid-state PEDOT:PSS-based vertical OECTs. As depicted in fig. S3, the ions cannot be driven into the PEDOT:PSS channel under a low gate voltage (i.e., 2.5 V), resulting in negligible modulation of the drain current. However, applying a relatively high gate voltage [i.e., 3 V, Fig. 1J (ii)] causes serious deformation of the planar drain electrode and leads to device breakdown due to the forced massive penetration of the polyelectrolyte under high electric field. Alternatively, a hole was opened in the planar Au drain, designed to function as a top-frame drain while also enabling sufficient direct contact between pDADMAC and PEDOT:PSS (Fig. 1A). As shown in Fig. 1K (i) initially, the electrolyte gating effect is weak, as indicated by a small *I-V* hysteresis loop, and then the hysteresis gradually opens up with subsequent *V*_G_ sweeps, eventually providing a memory window of ~2 V and an on/off ratio of six orders of magnitude. This transition originates from the cationic pDADMAC, which is blocked by the top-frame drain and requires a certain period to penetrate into the overlapped PEDOT:PSS region, gradually inducing a deep electrochemical reaction (Fig. 1G). This dynamic behavior is analogous to the plasticity observed in synapses, where connections between neurons can be modulated over time in response to stimuli. When the frame drain is positioned under the channel (Fig. 1B), the OECT operates as a switching transistor with high on/off ratio and small hysteresis [Fig. 1K (ii)] because there is no ion transport barrier from the buried frame drain (Fig. 1H). In conclusion, the transport behaviors of the polyelectrolyte pDADMAC and hence the performance of solid-state vertical OECTs can be effectively manipulated, which provides a promising avenue for various applications.

### Monolithic fabrication and electrochemical reaction mechanism

**Fig. 2.**
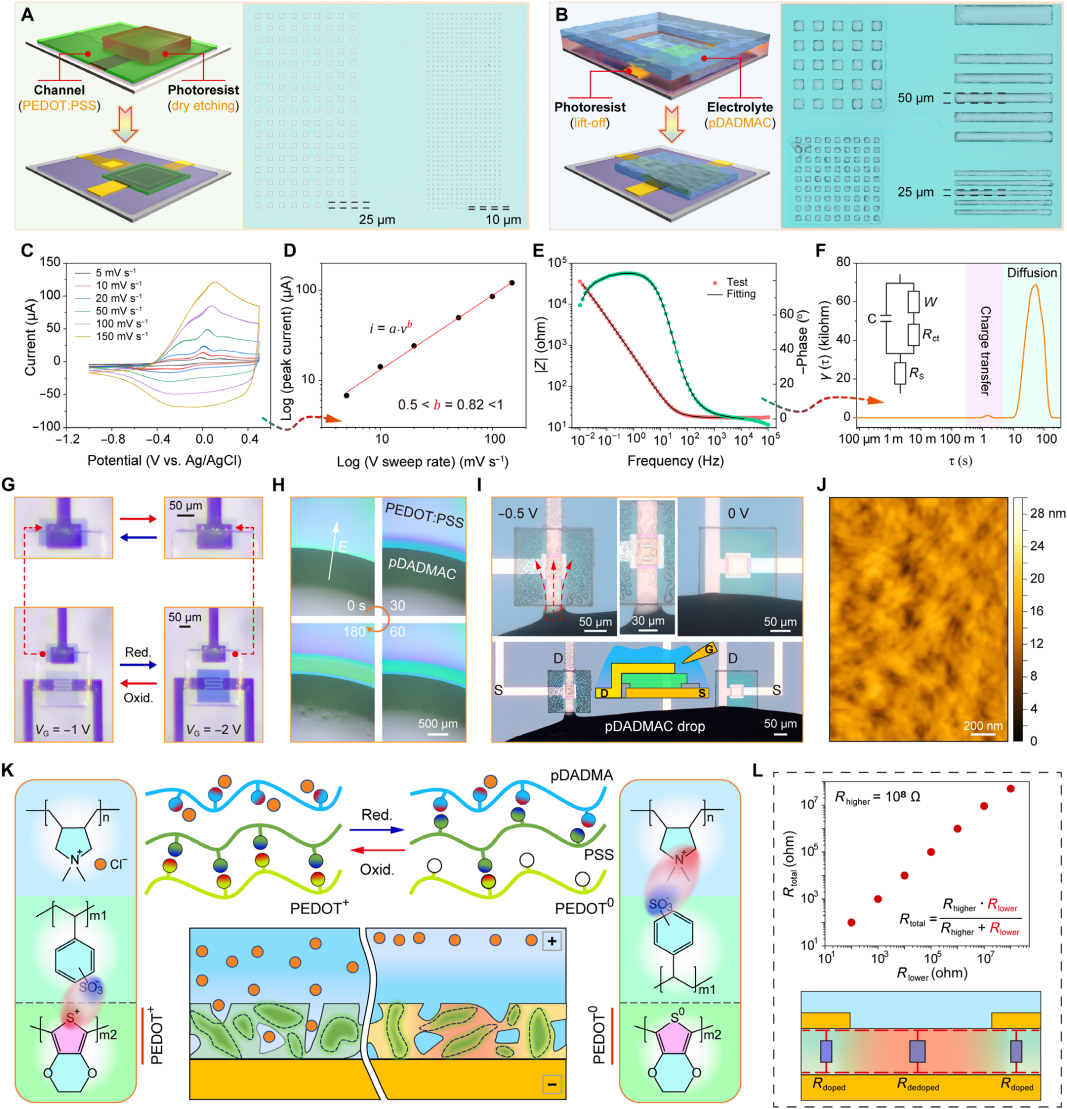
Monolithic fabrication and electrochemical reaction mechanism. (**A**) Patterning process of PEDOT:PSS film via O_2_-plasma etching process and corresponding optical image. (**B**) Patterning process of pDADMAC film via lift-off process and corresponding optical image. (**C** and **D**) Sweep rate–dependent CV and the corresponding *b*-value fitting. (**E**) EIS. (**F**) DRT analysis based on EIS data shown in (E). Inset is the equivalent circuit, where *R*_s_, *R*_ct_, *C*, and *W* are electrolyte resistance, charge transfer resistance, capacitance, and Warburg impedance, respectively. (**G**) Electrochromic behaviour of lateral OECT based on PEDOT:PSS gate (*V*_D_ = −0.5 V). (**H**) Movement of polycations under −10 V. (**I**) Controllable movement of polycations along the negatively charged drain line (left) and non-charged drain line (right). (**J**) Atomic force microscopy image of the porous PEDOT:PSS film. (**K**) Proposed electrochemical reaction mechanism between solid-state pDADMAC and porous PEDOT:PSS. pDADMAC has positively charged ammonium groups and PSS has negatively charged sulfonate groups. (**L**) Total resistance of the PEDOT:PSS channel with top frame is governed by the overlapped region, which is less dedoped and has low resistance compared to the exposed region (bottom). Relationship between the total resistance and lower resistance in a parallel circuit, where the higher resistance is set at 10^8^ ohm (top).

The utilization of solid-state electrolytes is essential for OECTs to achieve high-density integration, flexible electronic systems, and individual manipulation of each OECT cell (*23*). However, the amphoteric nature of PEODT:PSS poses challenges to commonly used acidic photoresists and alkaline developers, leading to serious contamination risks (fig. S4), while pDADMAC’s high water solubility means that the water from either the photoresist or developer can potentially destroy the patterned structures (fig. S5). To address these issues, a water-free photoresist AR-P 5910 with a neutral pH was used here to pattern pDADMAC film via a lift-off process (Fig. 2A) and to etch the uncovered PEDOT:PSS film by O_2_ plasma (Fig. 2B). PEDOT:PSS patterns with feature sizes down to 10 μm and pDADMAC patterns as small as 25 μm were successfully achieved (fig. S6).

As demonstrated above, the patterned pDADMAC electrolyte can effectively modulate the PEDOT:PSS channel, delivering superior performance and benefit of monolithic fabrication compared to the patterned solid-state electrolytes based on poly(vinyl alcohol) (PVA) and poly(acrylic acid) (PAA) that we prepared (fig. S7 and note S2). The sweeping rate-dependent cyclic voltammetry (CV; Fig. 2C) confirms the occurrence of an electrochemical reaction between PEDOT:PSS and pDADMAC, characterized as a solid diffusion-dominated process as implied by the fitted *b* value (0.5 < *b* = 0.82 < 1; Fig. 2D). The presence of Warburg impedance (*W*) in the electrochemical impedance spectroscopy (EIS; Fig. 2E)–based equivalent circuit, along with the solid-diffusion peak observed in the distribution of relaxation times (DRT; Fig. 2F and note S3) analysis (*24*), provides additional evidence for a bulk electrochemical reaction within PEDOT:PSS, rather than a mere surface reaction. These phenomena indicate that the ions from pDADMAC can permeate into the body of PEDOT:PSS, which is further supported by the electrochromic performance observed in lateral OECTs (Fig. 2G and movie S1).

Considering the large size of pDADMAC polycations compared to small ions such as Na^+^, it is difficult to imagine that they are able to be transported so easily. A collection of investigations indicates that polycations in pDADMAC are mobile under the electric field (Fig. 2, H and I and note S4). At the same time, the porous nature of the PEDOT:PSS film (Fig. 2J) and the low average molecular weight of the pDADMAC electrolyte enable internal diffusion. Notably, both pDADMAC and PEDOT:PSS are water-based solutions, allowing the pDADMAC solution to infiltrate into the porous PEDOT:PSS film before spin coating (fig. S11 and movie S4) (*25*, *26*), thereby further promoting the penetration. The electrostatic interaction between pDADMAC and PSS has been widely used to construct layer-by-layer self-assembled polyelectrolyte multilayers (*27*, *28*). Their intimate interaction led to delamination of the pDADMAC patterns in vacuum, which directly tore the underlying PEDOT:PSS film (fig. S12). Compared to a bare PEDOT:PSS film, the conductivity of the solid-state pDADMAC-covered PEDOT:PSS film decreased (fig. S13), possibly due to the partial dedoping of PEDOT component caused by the interaction between pDADMAC and PSS.

On the basis of the above discussion, the gating process can be explained as follows (Fig. 2K): Polycations from pDADMAC (located within and surrounding the porous PEDOT:PSS film) interact electrostatically with PSS polyanions under the influence of the gate voltage, resulting in the deep dedoping of the charged PEDOT and the consequent reduction in drain current. For bottom-frame drain-based solid-state vertical OECTs (BF-*v*OECT), there is no need for a stabilization process because the interdiffusion has already occurred during fabrication [Fig. 1K (ii)]. However, the top-frame drains of the vertical OECTs (i.e., TF-*v*OECTs) serve as ion blockers, which can divide the channel into doped (low resistance) and dedoped (high resistance) regions at the initial stage of applying a gate voltage (Fig. 2I, down). In paralleled resistors, the total resistance is always dominated by the smallest resistor in the circuit (Fig. 2I, top). Therefore, in the top-frame drain-based vertical OECTs, the drain current can only be completely turned off when the pDADMAC electrolyte penetrates into the overlapping PEDOT:PSS region and dedopes this part of PEDOT cations [Fig. 1K (i)].

### Behaviors of multilevel synaptic-OECTs

**Fig. 3.**
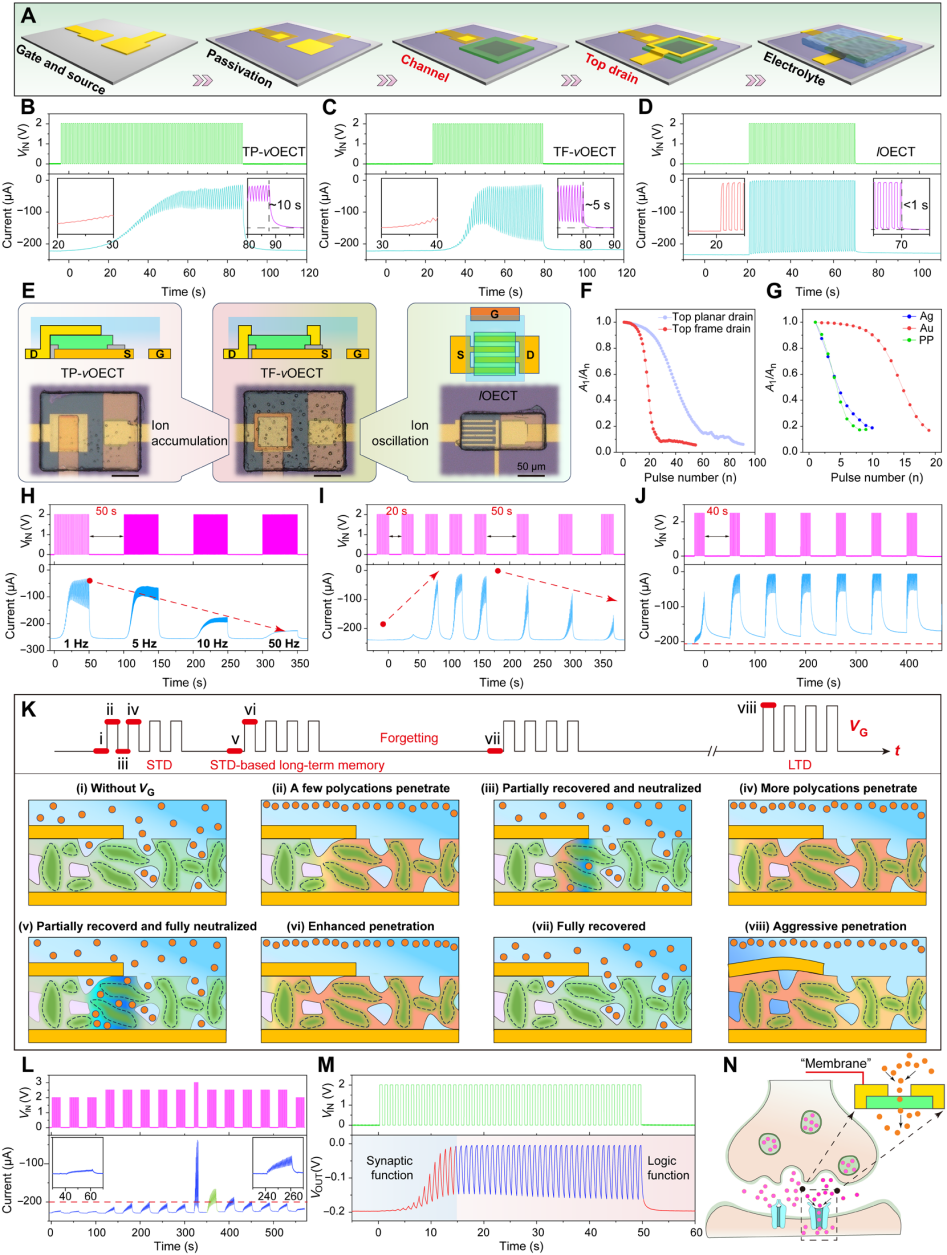
Synaptic behaviors of integrated solid-state vertical OECTs based on top drains. (**A**) Integration process for solid-state TF-*v*OECTs. (**B** to **D**) Plasticity performances of (B) TP-*v*OECTs, (C) TF-*v*OECTs, and (D) *l*OECTs. Upon applying multiple spikes (1 Hz), the PSCs are progressively inhibited step by step, resulting in distinct conductance states (see insets). The enlarged versions of (B) to (D) are provided in fig. S19 for better clarity. (**E**) *I*_D_ behavior of TF-*v*OECTs demonstrates a combination or transition state between TP-*v*OECTs and *l*OECTs. The optical images are top-plane drain, top-frame drain, and lateral OECTs, respectively. (**F**) Inhibitory index (*A*_n_/*A*_1_) of synaptic OECTs based on top-plane and top-frame drains as a function of pulse number. (**G**) Inhibitory index (*A*_n_/*A*_1_) of TF-*v*OECTs based on Ag, Au, and PEDOT:PSS gate materials as a function of pulse number. (**H**) Frequency-dependent plasticity of TF-*v*OECTs with spike (0 → 2 V) frequencies ranging from 1, 5, 10, and 50 Hz. (**I**) STD-based long-term memory (short time interval, left) and forget (long time interval, right) at 1 Hz. (**J**) LTD triggered by high presynaptic voltages at 1 Hz. The enlarged versions of (H) to (J) are provided in fig. S20 [(A) to (C)] for better clarity. (**K**) Modulation mechanism of STD, STD-based long-term memory, and LTD by the decoupling between the redoping of PEDOT^0^ and the electrical neutralization of polycations with Cl^−^ anions upon the removal of Vg. (**L**) Voltage-dependent training-learning behavior of TF-*v*OECTs based on PEDOT:PSS gate at 1 Hz. The enlarged version is provided in fig. S20D for better clarity. (**M**) Seamless transition between synaptic function and logic function as the pulse number increases at 1 Hz. (**N**) Top-frame drain acts as the postsynaptic membrane. For all the measurements, *V*_D_ = −0.2 V and PEDOT:PSS thickness = ~30 nm.

Integrating a drain electrode on the channel (Fig. 3A) creates a physical barrier to electrolyte transport, enabling the vertical OECT to mimic a biological synapse. As shown in Fig. 3 (B to D), it is obvious that (i) both top planar drain (TP)–*v*OECTs and TF-*v*OECTs exhibit a gradual decay in the drain current *I*_D_ [analogous to the postsynaptic current (PSC)] triggered by consecutive presynaptic spikes (Fig. 3, B and C), whereas (ii) their lateral counterparts (i.e., *l*OECTs) display a switching response with negligible attenuation (Fig. 3D). This notable difference directly proves that the top drains can restrict electrolyte penetration. The PSC behavior of TF-*v*OECTs resembles a combination or transition state between the TP-*v*OECTs and *l*OECTs (Fig. 3E): The gradual decrease of PSC at the beginning (left in Fig. 3C) has the same tendency seen in Fig. 3B, and the subsequent switching-like response (right) is similar to that of *l*OECT (Fig. 3D). The current loss ratio, defined as *A*_n_/*A*_1_ (*A*_n_ represents the peak current of the nth spike), is plotted in Fig. 3F to reflect how effectively the synaptic OECTs attenuate the signals. Compared to TP-*v*OECTs, TF-*v*OECTs require fewer activation spikes (approximately 10) and exhibit sharper attenuation in PSC because the frame drain opens a larger window for electrolyte diffusion while the narrower frame width facilitates easier ion transport (Fig. 3E). In contrast, the channel in *l*OECT is fully exposed to electrolyte, leading to reversible switching. The structure modulation also affects the recovery response, with recovery times of about 10 s (Fig. 3B), 5 s (Fig. 3C), and 0.8 s (Fig. 3D). In addition, the nature of gate electrodes affects the ion transport efficiency (note S5). As depicted in Fig. 3G, gates based on Ag and PEDOT:PSS exhibit faster modulation compared to those based on Au gates.

Figure 3H and fig. S15 illustrate the spike frequency-dependent modulation, where the duty cycle is kept at 50%. Under this circumstance, higher frequencies correspond to smaller changes in PSC amplitudes and reduced fluctuations in the saturated PSCs. This occurs because at high frequency, short spike duration limits ion penetration (small PSC change), while short time interval restricts ion recovery (small fluctuation). All the above-described synaptic behaviors follow the pattern of STD (*29*), reflecting transient adjustments in synaptic strength and a temporal memory effect (*30*, *31*). However, long-term memory-like behavior can occur in the TF-*v*OECTs among multiple-STD training. As shown in Fig. 3I and fig. S16, each STD set is triggered by 20 consecutive presynaptic spikes (1 Hz, 2 V). For the first spike group, the PSC change is negligible, but it then decreases progressively during the subsequent groups until reaching a stable level by the fourth spike group. By extending the time interval between groups from 20 to 50 s, the memory effect diminishes and the modulation amplitude in each group becomes weaker and weaker.

A question arises: How does the long-term cumulative effect come about even when the PSCs have recovered to their initial level after every spike group (i.e., STD)? We attribute this phenomenon to the decoupling between the redoping of PEDOT^0^ and the electrical neutralization of polycations with Cl^−^ anions when switching off the gate voltage (*V*_G_). Within one spike group, polycations gradually migrate into the covered PEDOT:PSS channel and electrostatically interact with PSS^−^ polyanions, consequently dedoping PEDOT^+^ [Fig. 3K (i and ii)]. The top drain impedes the full recovery of the large-size polycations over a short time (e.g., 0.5 s), but small Cl^−^ anions from the electrolyte can move relatively freely to partially neutralize the polycations. Therefore, a part of the dedoped PEDOT segments is retained [Fig. 3K (iii)], facilitating accumulated inhibition for the following spikes [Fig. 3K (iv)]. However, when the time interval between spike groups is 20 s, which is not long enough for polycations to fully recover but allows sufficient time for the Cl^−^ anions to completely neutralize the trapped polycations [Fig. 3K (v)], thus facilitating the full recovery of PSC and resulting in STD. Therefore, the accumulation of partially trapped polycations during the previous presynaptic spike group leads to greater PSC suppression in the subsequent groups [Fig. 3K (vi) and Fig. 3I, left]. When the time interval between spike groups is further extended, such as to 50 s, the trapped polycations can diffuse back slowly due to the concentration gradient [Fig. 3K (vii)], resulting in gradually weakened cumulative effect and reduced PSC change (Fig. 3I, right). In Fig. 3K, the orange region represents reduced PEDOT:PSS, indicating areas where pDADMAC has successfully penetrated; the dark blue region indicates nonrecovered pDADMAC beneath the top drain, highlighting areas where the penetrated pDADMAC cannot fully exit within a short time.

The long-term-like memory resulting from closely spaced STD behaviors is not the traditional manner of long-term synaptic plasticity (*8*). We find that increasing the presynaptic spike from 2 to 2.5 V prevents the inhibited PSCs from recovering to the baseline, and the shift amplitudes become notable with the subsequent spike groups (1 Hz, 10 cycles, 40-s interval; Fig. 3J), indicating a successful transition from STD to LTD. This transition is forcibly triggered by the intense and deep penetration in a short time under high gate voltages [Fig. 3K (viii)], accompanied by morphological changes observed beneath the top drain region (fig. S17).

The synaptic OECTs can exhibit voltage-dependent training-learning behavior, as shown in Fig. 3L. At low-voltage levels (e.g., 2 and 2.5 V), small PSC changes with slight variations occur in each spike group (1 Hz, 10 cycles), while introducing higher voltage (3 V) for a short period triggers a substantial increase in PSC change, which causes even larger PSC changes for the subsequent spike groups than previous ones at the same voltage (2 V), and then gradually returning to the same change amplitude in the successive groups. This behavior suggests a form of activity-dependent plasticity, reflecting Hebbian principles (*32*). Moreover, TF-*v*OECTs combine the features of TP-*v*OECTs and *l*OECTs, enabling a seamless transition from synaptic OECT (with gradually inhibited synaptic strength) to logic OECT (characterized by reversible switching) in a standard inverter circuit (Fig. 3M and fig. S18). In summary, the polycations function similarly to inhibitory neurotransmitters such as γ-aminobutyric acid (GABA) (*33*) in the synaptic OECTs, regulating the synaptic weight through the top drains (Fig. 3N).

**Fig. 4.**
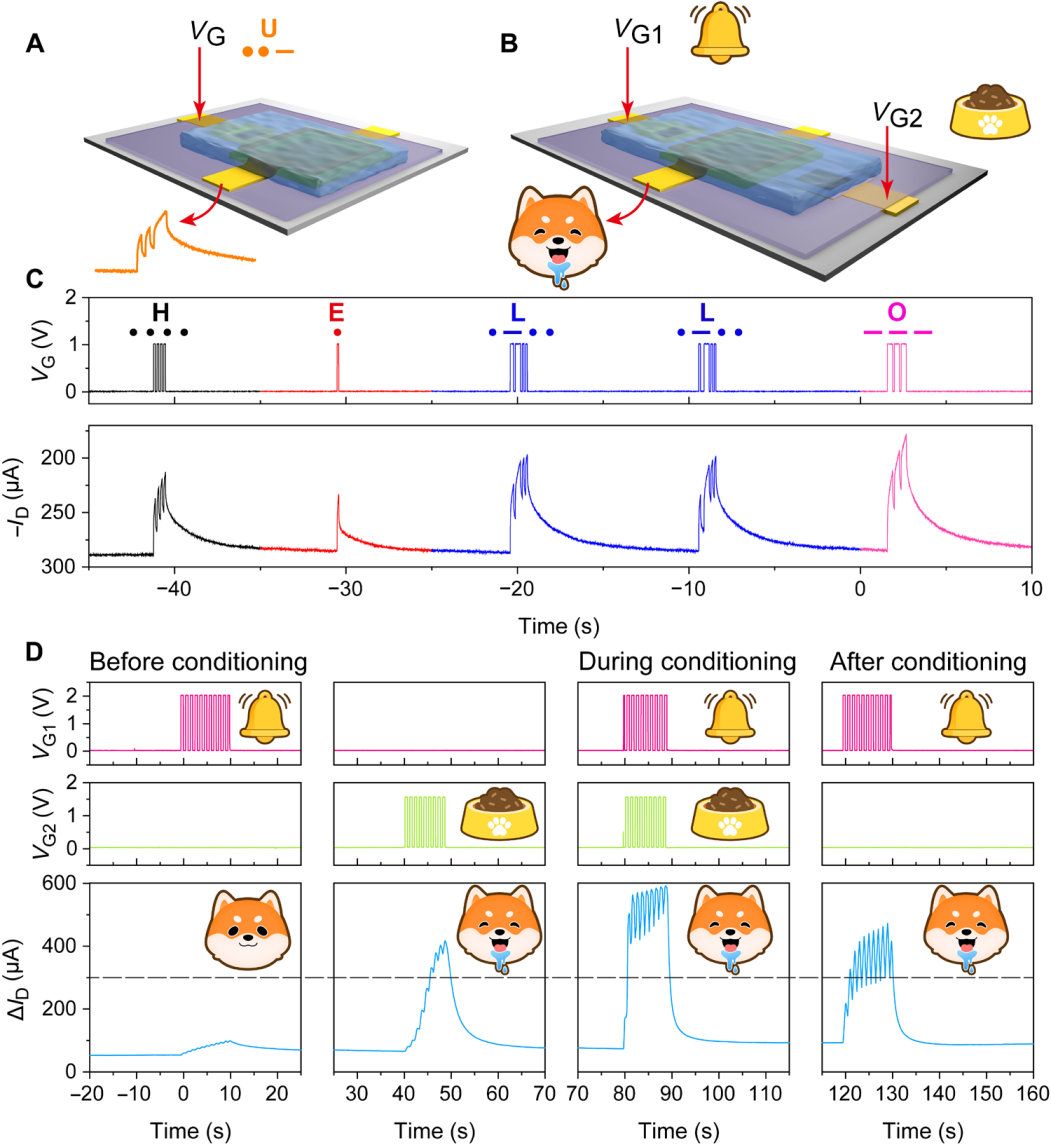
Applications of top-frame vertical OECTs in encrypted communication and Pavlovian associative learning. (**A** and **C**) International Morse code for “HELLO” realized using TF-*v*OECT. (**B** and **D**) Pavlovian learning behaviour realized using TF-*v*OECT.

We demonstrate the potential applications of our synaptic TF-*v*OECTs in encrypted communication using international Morse codes (Fig. 4A) and Pavlovian associative learning (Fig. 4B). In Morse code communication, each letter of the English alphabet comprises a unique combination of “dots (.)” and “dashes (−)”, encoded here by short-duration (1 V, 100 ms) and long-duration (1 V, 300 ms) spikes, respectively. Using this approach, the input letters “HELLO” were successfully encoded and transmitted (as shown in Fig. 4C), which generated a distinct amplitude response corresponding to different letters through short-term memory. This demonstration highlights the system’s high accuracy and temporal robustness in encoding and processing encrypted information.

In addition, dual-gate TF-*v*OECTs were integrated to conduct the famous Pavlov’s dog experiment, also known as an example of associative learning. As depicted in Fig. 4D, presynaptic electrical spikes applied to Gate 1 (Au) and Gate 2 (Ag) were regarded as “ring bell/neutral stimulus” and “feeding food/unconditioned stimulus”, respectively, while the change in *I*_D_ served as the output neuron, corresponding to the “salivation” response. Before training, 10 consecutive ringing “bell” stimuli (2 V via Au gate) failed to induce salivation. Then, salivation was evoked by 10 consecutive “food” stimuli (1.5 V via Ag gate). During training, the bell stimuli and “food” stimuli were applied simultaneously, which caused a larger *I*_D_ change compared to previous individual stimuli, indicating the formation of an associative reflex between the conditioned and unconditioned stimuli. Following training, the bell stimulus alone was sufficient to evoke an *I*_D_ change above the salivation threshold, successfully mimicking the salivation response of Pavlov’s dog to bell ringing stimuli.

### Behaviors of monolithic logic OECTs

**Fig. 5.**
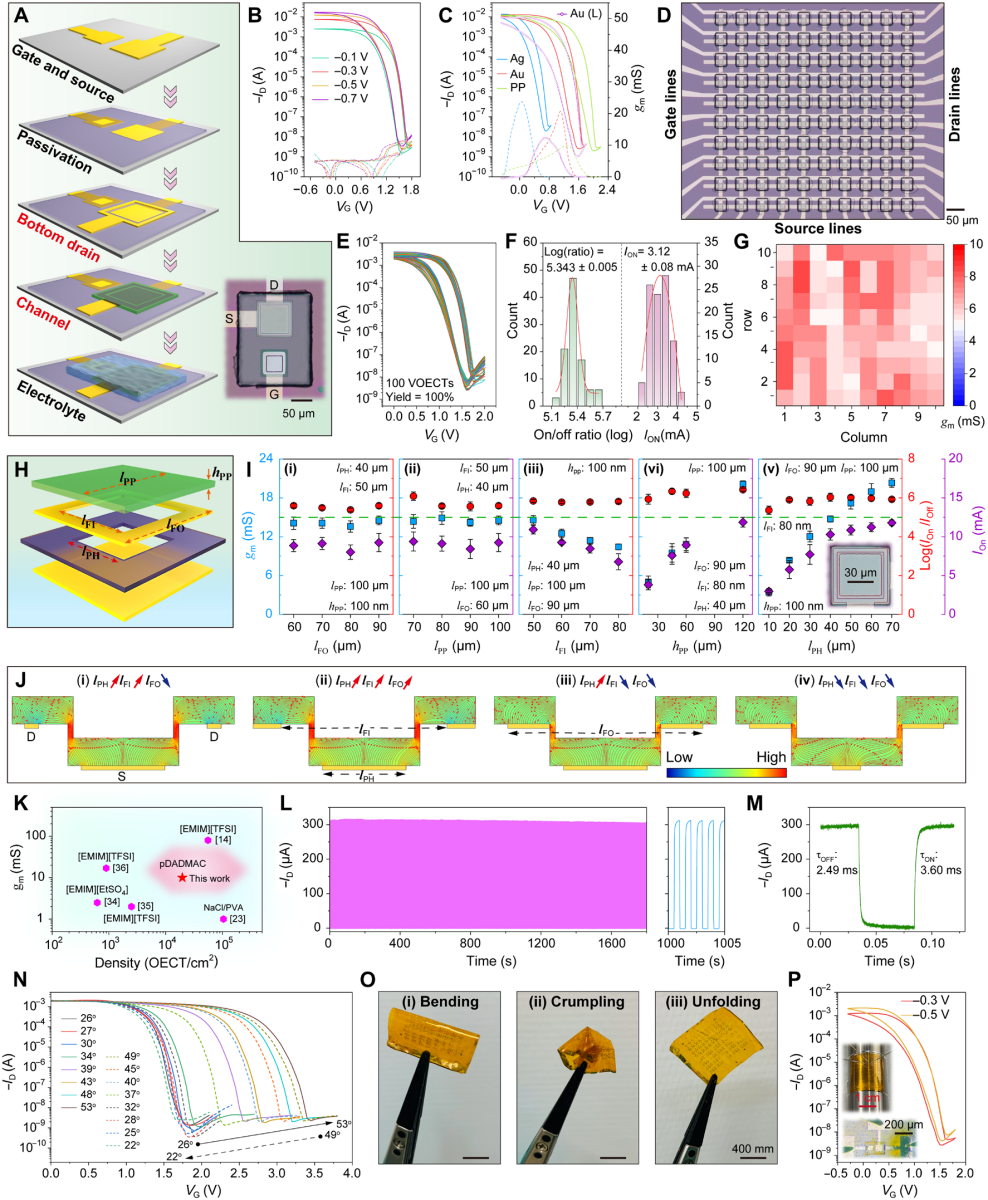
Logic behaviors of integrated solid-state vertical OECTs based on bottom drains. (**A**) Integration process for solid-state BF-*v*OECTs. (**B**) Representative transfer characteristics of BF-*v*OECTs as a function of drain voltages. (**C**) Representative transfer characteristics of BF-*v*OECTs based on Ag, Au, and PEDOT:PSS gate materials, as well as lateral OECTs with Au gate for comparison. There corresponding *g*_m_ curves are also shown (dotted lines). (**D**) Optical image of a 10 × 10 solid-state BF-*v*OECTs. PEDOT:PSS thickness in (B) to (D) is ~30 nm. (**E**) Transfer characteristics of 100 BF-*v*OECTs. (**F**) Statistical distribution histograms of on/off ratio (left) and on-current (*I*_ON_, right) for 100 devices, which are all functioning. (**G**) Transconductance distribution in the 10 × 10 solid-state BF-*v*OECTs. (**H**) Definitions of the key structural parameters of the BF-*v*OECTs, including the size of the passivation square hole (*l*_PH_), the inner and outer sizes of the bottom square frame (*l*_FI_ and *l*_FO_), as well as the size and thickness of the PEDOT:PSS square pattern (*l*_PP_ and *h*_PP_). (**I**) Statistics of *g*_m_, on/off ratio, and on-current based on *l*_FO_ (i), *l*_PP_ (ii), *l*_FI_ (iii), *h*_PP_ (iv), and *l*_PH_ (v). (**J**) Simulated electric field distributions of various configurations. (**K**) Comparison of integration density versus *g*_m_ between our polyelectrolyte-based BF-*v*OECTs and reported integrated solid-state OECTs, which are based on small ions. (**L**) Cycling stability (frequency of 1 Hz) of the BF-*v*OECTs and the corresponding magnification curve (left), where *V*_D_ = −0.3 V. (**M**) Transient response of the BF-*v*OECTs (*V*_D_ = −0.3 V). (**N**) Temperature-dependent transfer characteristics of BF-*v*OECTs. (**O**) Flexible OECT arrays under different mechanical deformation modes. (**P**) Representative transfer characteristics of the flexible BF-*v*OECTs after deformation. PEDOT:PSS thickness: ~30 nm. The insets show measurement setup for the bent OECTs and a bent OECT being test.

When the frame drain is positioned below the PEDOT:PSS channel (Fig. 5A), the devices act as logic OECTs. Representative transfer curves shown in Fig. 5B and fig. S21 indicate typical depletion-mode p-type characteristics, achieving a maximum drain current (*I*_ON_) of −1.7 × 10^−2^ A (*V*_D_ = −0.7 V, *V*_G_ = −0.5 V) and an on/off ratio of six orders of magnitude (between −0.5 to 1.8 V). In addition, by modulating the gate materials (Fig. 5C), the threshold voltages (*V*_th_) can be effectively controlled from 0.7 V (Ag) to 1.6 V (Au) to 2 V (PEDOT:PSS), with corresponding transconductance (*g*_m_ = ∂*I*_D_/∂*V*_G_) values of 23.7, 20.6, and 11.1 mS, respectively. Compared to vertical OECTs, the lateral counterparts with Au gates exhibit smaller *g*_m_ (12.3 mS) and larger hysteresis (Fig. 5C), highlighting the superior performance of the bottom-frame drain design. To access scalability and reliability, we fabricated a monolithically integrated solid-state vertical OECT array with a pDADMAC pattern size of 30 μm by 40 μm (fig. S22), which contains 100 transistors within a 0.507-mm^2^ substrate area (Fig. 5D), translating into a device density of ~19,700 transistors/cm^2^. The corresponding transfer curves of these 100 OECTs are shown in Fig. 5E, demonstrating highly uniform performance with a yield of 100%. Statistics on the devices’ on/off ratio (in logarithmic form, between 0 and 2 V), *I*_ON_, and *g*_m_ are presented in Fig. 5 (F and G), showing values of 5.344 ± 0.004, 3.125 ± 0.081 mA, and 6.5 ± 1.0 mS, respectively.

As presented in Fig. 5 (B and E), the performance of the vertical OECTs is closely related to their feature sizes. Therefore, several critical dimensional parameters are defined in Fig. 5H, and their impacts on the performance are elaborated in note S6, supported by statistical data (Fig. 5I) and simulated electric field distributions (Fig. 5J). Note that the electric field in the vertical configuration is a hybrid of both vertical and lateral modes. Consequently, the positioning of the frame drain and source electrodes greatly influences the electric field distribution and hole transport within the channel, presenting opportunities to optimize device performance. A comparison in Fig. 5K illustrates that our monolithically fabricated polycation-based OECTs exhibit not only good integration density but also high *g*_m_, comparable to the outstanding small ion–based solid-sated OECTs reported previously (*14*, *23*, *34*–*36*).

The cycling stability and the transient response are shown in Fig. 5L. More than 1000 stable switching cycles (1 Hz) are recorded with negligible performance degradation. The turn-on and turn-off transient times (τ_ON_ and τ_OFF_) are a few milliseconds (Fig. 5M), shorter than those of their lateral counterparts (fig. S23). After being stored in air for 225 days without any encapsulation, the BF-*v*OECTs exhibited negligible performance attenuation (fig. S24), underlining their exceptional durability and reliability under ambient conditions. Moreover, our BF-*v*OECTs exhibit an opposite temperature trend to that of small ion–based OECTs (*37*), with threshold voltages rising from 1.6 V to 3.4 V as the temperature increased from 26° to 53°C (Fig. 5N and fig. S25). This abnormal behavior may stem from the thermal expansion of the polycation electrolyte, which reduces ionic conductivity in the PEDOT:PSS channel, necessitating a higher gate voltage for sufficient polycation attraction to facilitate the electrochemical reaction. The *V*th returned to its initial level as the temperature dropped from 53° to 22°C and showed slight variation between 22° and 34°C, indicating thermal reversibility and stability. On the other hand, our solid-state OECTs still operate reliably in acetone (fig. S26, A and B, and movie S5), critical for applications in challenging environments. Also, the noncrosslinked pDADMAC also functioned reliably in water when encapsulated with PDMS (fig. S26, C to E, and movie S6). Given the high flexibility of polymers such as PEDOT:PSS and pDADMAC, we successfully integrated OECTs on a polyimide (PI) substrate (Fig. 1E and fig. S27). Figure 5O displays various mechanical deformations of the flexible OECT array, including bending, crumpling, and re-expanding (fig. S28). Following these deformations, testing the flexible OECTs bent around a rod (Fig. 5P) shows that they maintain performance comparable to those on silicon substrates [Fig. 1K (ii)], highlighting the robustness and adaptability of our flexible electronics platform.

**Fig. 6.**
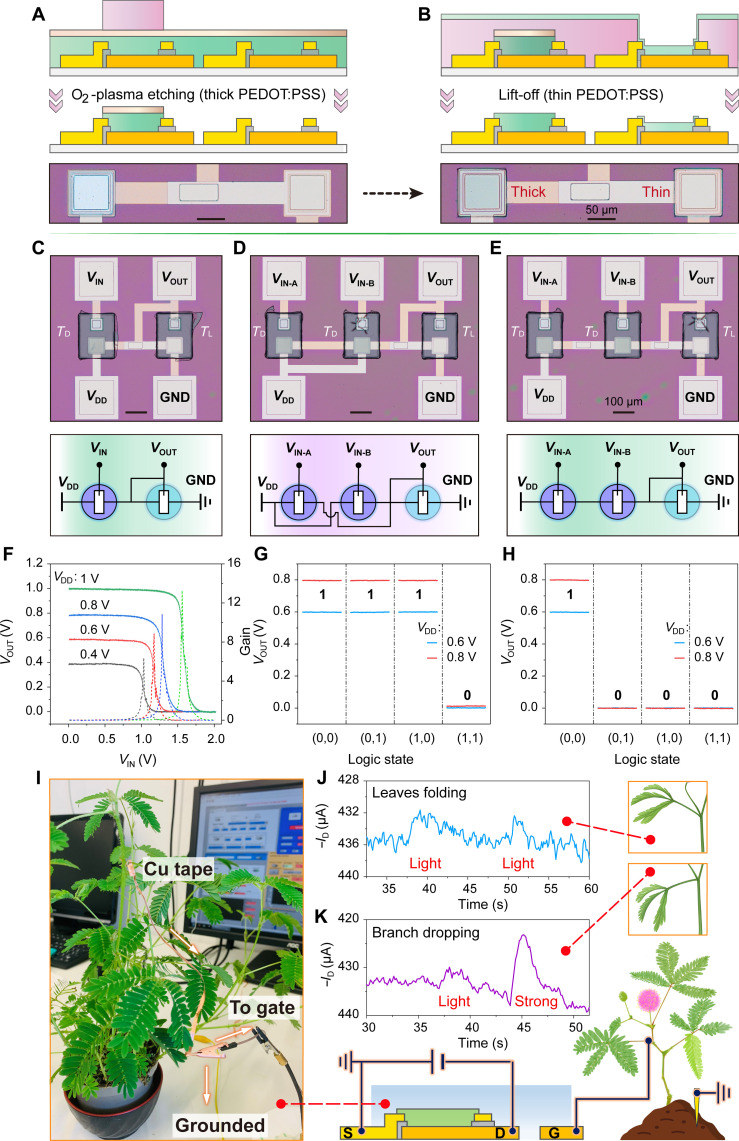
Applications of bottom-frame vertical OECTs. (**A** and **B**) Fabrication processes of BF-*v*OECT-based logic circuits: thick PEDOT:PSS channel via O_2_-plasma etching process (A) and thin PEDOT:PSS channel via lift-off process (B). (**C** to **E**) Optical images and the corresponding circuit schematics of inverter (C), NAND (D), and NOR (E) based on solid-state BF-*v*OECTs. *T*_D_ and *T*_L_ represent drive and load transistors, respectively. (**F**) Representative voltage output characteristics of the bottom-frame OECT-based inverter with the voltage gain. (**G** and **H**) Output characteristics of the NAND (G) and NOR (H) logic circuits. The NAND gate provides a logic value of 0 when both inputs are 1 and the NOR gate exhibits a logic value of 1 only when both inputs are 0. (**I**) Optical photo and circuit diagram used for signal recording. (**J** and **K**) Recorded electrophysiological signals from *M. pudica* upon triggering the leaves (J) and branch (K) with the BF-*v*OECT receptor, *V*_D_ = −0.1 V.

Next, solid-state logic circuits were constructed by integrating the BF-*v*OECTs into unipolar inverters, NAND, and NOR gates, where the driver transistor(s) were connected in series with a load transistor. To ensure that the load transistor can take most of the voltage drop before activating the driver transistor(s), we developed a monolithic process to integrate OECTs with different PEDOT:PSS thicknesses on the same chip (Fig. 6, A and B, and fig. S29), further demonstrating the viability of our monolithic fabrication process. Figure 6F presents the voltage output characteristics of the unipolar zero-*V*_G_ load inverter (Fig. 6C) at different applied voltages. The dotted lines in Fig. 6F represent the calculated signal gain, defined as |d*V*_OUT_/d*V*_IN_|, which is determined to be approximately 14 at *V*_DD_ = 1 V. For the NAND and NOR gates, two driver transistors are configured in parallel and series (Fig. 6, D and E), respectively, and then connected serially to the load transistor. Input voltages of 1.5 and 0 V correspond to logic states “1” and “0.” Both NAND and NOR gates demonstrate well-defined Boolean outputs with *V*_DD_ = 0.6 and 0.8 V, as shown in Fig. 6 (G and H). Therefore, all four input logic combinations [(0,0), (1,0), (0,1), and (1,1)] are successfully processed by our gates. We further examined the dynamic behaviors of these logic circuits (fig. S30, A and C), all of which demonstrated correct rail-to-rail responses. However, because of the inherent properties of the large polyelectrolyte and the multiple OECT connections, the output voltage fails to perfectly reach zero in the “0” state at high frequencies (fig. S30B).

Furthermore, BF-*v*OECTs were used to record the electrical impulses generated by sensitive *Mimosa pudica*. A copper tape was used to connect the OECT gate to the *Mimosa* branch (Fig. 6I and movie S7), with the *Mimosa* action potential serving as the gate input and *V*_D_ = −0.1 V. A light touch on the leaves resulted in subtle variations in the recorded *I*_D_, corresponding to the folding of the leaves (Fig. 6J). In contrast, a stronger touch caused the branch to drop, resulting in a notable change in *I*_D_ (Fig. 6K).

## DISCUSSION

We have developed a monolithic integration process for high-density solid-state vertical OECTs on both rigid and flexible substrates. Compared to small ions such as Na^+^, the polyelectrolyte pDADMAC provides superior control over ion transport within the PEDOT:PSS channel. These large ions interact with the channel in more complex ways, enabling advanced manipulation of OECT functionalities. By carefully designing the drain electrodes and mastering the polycations, our integrated solid-state OECTs can operate between neuromorphic and logic functions. Our approach is expected to expand the manipulation of ionic-electronic coupling and enhance the functionality of OECTs.

## MATERIALS AND METHODS

### Materials

pDADMAC (average molecular weight < 1000,000, classified as “very low–molecular weight” by the supplier, 35 weight % in H_2_O) as the electrolyte was purchased from Sigma-Aldrich. Aqueous PEDOT:PSS (Clevios F HC) and PEDOT:PSS:AgNWs (Clevios HY E), purchased from Heraeus, served as the organic channel and top drain electrodes. Photoresist AR-P 5910 purchased from Allresist GmbH was applied to pattern pDADMAC and PEDOT:PSS films. Photoresist AZ 5214E purchased from Microchemicals GmbH was used for metal lift-off processes. Photoresist SU-8 2000.5 purchased from Microchemicals GmbH was used for metal lift-off processes.

### Patterning of PEDOT:PSS film

The following steps were performed for the patterning of PEDOT:PSS film: (i) Aqueous PEDOT:PSS was spin-coated at different speeds to achieve certain film thicknesses. (ii) The spin-coated PEDOT:PSS film was dried at 90°C for 10 min. (iii) AR-P 5910 was spin-coated on top of the PEDOT:PSS film and was patterned via a photomask. (iv) The exposed PEDOT:PSS was etched for 120 s by oxygen plasma at 400 W. (v) Patterned AR-P 5910 was removed in acetone, rinsed with deionized water, and then dried with N_2_. To improve the adhesion between PEDOT:PSS and AR-P 5910 to obtain higher pattern resolution, a layer of Ag film can be deposited before the spin coating of 5910. The unwanted Ag region defined by the AR-P 5910 pattern was etched by a mixed acidic solution, H_3_PO_4_:HNO_3_:CH_3_COOH:H_2_O (in a ratio of 3:3:23:1). Afterward, the PEDOT:PSS layer was exposed, which can be easily removed by O_2_ plasma.

### Patterning of pDADMAC film

The following steps were performed for the patterning of pDADMAC film: (i) AR-P 5910 was spin-coated at 2000 rpm and then patterned via a photomask to create grooves. (ii) pDADMAC was spin-coated on top of the AR-P 5910 pattern and then dried naturally. (iii) The unwanted pDADMAC was removed by lifting off the AR-P 5910 in acetone. (iv) The patterned pDADMAC was dried at 45°C.

### Fabrication of top drain-based vertical OECT array

The following steps were performed for the fabrication of top drain-based vertical OECTs: (i) 10 nm of Ti and 50 nm of Au were thermally evaporated as the bottom source and gate electrodes and patterned through AZ 5214E-based lift-off process. (ii) Passivation layer consisting of spin-coated SU-8 2000.5 or ALD-deposited Al_2_O_3_ was patterned to open rooms for source and gate pads. (iii) PEDOT:PSS channels were patterned via the process described above. (iv) Top drains, including the planar and frame Au or PEDOT:PSS:AgNWs drains, were patterned by a lift-off process with the help of AR-P 5910. (v) Solid-state pDADMAC patterns were achieved by the process described above.

### Fabrication of bottom drain–based vertical OECT array and the crossbar

The fabrication steps of bottom drain–based vertical OECTs were similar with the top drain-based OECTs, except that after the passivation layer patterning, 10 nm of Ti and 30 nm of Au were thermally evaporated as the bottom-frame drains and patterned through AZ 5214E-based lift-off process. Subsequently, PEDOT:PSS and pDADMAC were patterned.

For the fabrication of bottom drain–based vertical OECT crossbar array: (i) Ti/Au source lines were patterned and passivated by ALD-Al_2_O_3_ film. (ii) Windows were opened by etching the unwanted Al_2_O_3_ in HF solution with the help of AZ 5214E patterns. (iii) Drain lines were thermally evaporated and patterned through AZ 5214E-based lift-off process. (iv) SU-8 2000.5 passivation layer was pattern via photolithography and holes were created. (v) Gate lines were patterned. (vi) PEDOT:PSS channel was patterned. (vii) pDADMAC solid-state electrolyte was patterned.

### Fabrication of flexible OECT array

The fabrication steps of flexible OECTs were similar to the OECT fabricated on silicon substrate, except that PI tape was attached to silicon wafer manually, and then source, gate, passivation layer, and frame drain were subsequently patterned as described above. To avoid the thermal deformation of the PI tape during the O_2_-plasma etching, PEDOT:PSS channel was patterned via AR-P 5910–assited lift-off process. After that, pDADMAC was patterned. Last, the flexible OECTs on the PI tape were manually peeled off from the silicon substrate.

### Fabrication of OECT-based unipolar integrated circuits

The unipolar integrated circuits consist of driver transistors and load transistors with different thicknesses of PEDOT:PSS channels. Specifically, for the driver transistors, the PEDOT:PSS patterns with a thickness of ~100 nm were selectively etched using O_2_ plasma, guided by the photoresist protrusion structures (Fig. 6A), while the channel thickness in the load transistors was only about 30 nm, formed through a lift-off process from the photoresist recessed structures (Fig. 6B).

### Electrical characterization

The electrical characterization of the OECTs was carried in ambient conditions. All the transfer characteristics were measured by Keithley 2636A connected to a probe station. For synaptic behaviors in synaptic OECTs, transient response, and logic circuit test in logic OECTS, the voltage pulse was generated by a Tektronix AFG 3252 arbitrary function generator, *V*_DD_ was supplied by Keithley 2636A, and the related voltage variation was monitored with Tektronix TDS 1002B oscilloscope. The voltage-current conversion was realized by a resistor in series (820 ohm). The temperature was controlled by a thermoelectric cooler placed under the OECT wafers, which was monitored by a digital infrared thermometer.

### Electrochemical characterization

The CV and EIS measurements were conducted using an electrochemical workstation (MULTIAUTOLAB/M101) equipped with a conventional three-electrode system, where Ag/AgCl served as the reference electrode, Pt as the counter electrode, and a gold electrode coated with PEDOT:PSS as the working electrode, all within a pDADMAC electrolyte. For the EIS measurements, the frequency of the ac oscillation ranged from 0.1 to 10^5^ Hz with a single sinusoidal signal of 10 mV.

### Electric field simulation

The simulation of the electric field distribution in various bottom frame–based vertical OECTs was conducted using COMSOL Multiphysics.

## Supplementary Materials

### The PDF file includes:

Notes S1 to S6 Figs. S1 to S30 Legends for movies S1 to S7 References
