# Supplementary material for: Monolithically integrated solid-state vertical organic electrochemical transistors switching between neuromorphic and logic functions
Source: Sci Adv. 2025 Mar 14;11(11):eadt5186. doi: 10.1126/sciadv.adt5186 (PMC11908494; doi:10.1126/sciadv.adt5186)
Supplement: Supplementary file 1 — Notes S1 to S6 Figs. S1 to S30 Legends for movies S1 to S7 References [file sciadv.adt5186_sm.pdf]

Science Advances
AAAS

Supplementary Materials for

Monolithically integrated solid-state vertical organic electrochemical transistors switching between neuromorphic and logic functions

Tianming Li *et al.*

Corresponding author: Tianming Li, litimi2017@gmail.com;
Vineeth Kumar Bandari, vineeth-kumar.bandari@main.tu-chemnitz.de;
Oliver G. Schmidt, oliver.schmidt@main.tu-chemnitz.de

Sci. Adv. **11**, eadt5186 (2025)
DOI: 10.1126/sciadv.adt5186

The PDF file includes:

Notes S1 to S6
Figs. S1 to S30
Legends for movies S1 to S7
References

Other Supplementary Material for this manuscript includes the following:

Movies S1 to S7

Supplementary Notes

Supplementary Note 1: Strategies for controlling the hysteretic behavior in OECTs

The hysteretic behavior is commonly attributed to inhibited ion movement within the channel, and several strategies have been developed to regulate the dynamics of small ions and then either minimize or enhance the hysteresis loop to meet specific requirements. Ions with larger hydrated radii, such as Mg^{2+} and Ca^{2+}, have been found to exhibit slower transient responses when compared to monovalent ions with smaller hydrated radii (like Na^{+} and K^{+}) (*11*). Conversely, altering the composition and/or morphology of organic channels to control ion movement can also profoundly impact the OECT performance. For instance, Ma et al. (1*2*) demonstrated that controllable anion doping in the amorphous and crystalline PTBT-p channels enables the OECT to operate as a volatile receptor and a non-volatile synapse, respectively. On the other hand, the short-term or long-term plasticity of poly(3-hexylthiophene) (P3HT)-based synaptic OECTs can be precisely modulated by selectively binding the cross-linking additive to the P3HT channel via photochemical reactions, hence restricting ion penetration through the organic channel (1*3*). These efforts play a pivotal role in expanding the versatility of OECT-based devices across diverse fields.

Supplementary Note 2: Patterned solid-state electrolytes

Poly (vinyl alcohol) (PVA)-based NaCl electrolyte gel was first dropped onto the OECT surface, yielding a typical depletion-mode transfer curve, as shown in Fig. S7A (orange line). After allowing the gel to dry overnight, the OECT remained operational but exhibited significant degradation in performance (Fig. S7A, blue line). To explore patterning possibilities, the electrolyte gel was spin-coated onto the photoresist mold. However, it could not be effectively patterned using the process we developed for pDADMAC (Fig. S7B-C). Thereafter, a photoinitiator ($K_2Cr_2O_7$) was added to the PVA gel to make the electrolyte patternable (Fig. S7D). Despite successful patterning, the resulted PVA electrolyte failed to effectively modulate the PEDOT:PSS channel (Fig. S7A, green line) until the additional salt solution was added (Fig. S7A, red line). Similarly, poly(acrylic acid) (PAA)-based electrolyte gel was prepared and patterned successfully (Fig. S7E); however, the device required a high gate voltage (~30 V) to achieve gating effects (Fig. S7F), limiting its practical application.

Following are the fabrication processes of PVA- and PAA-based electrolytes:

PVA-based electrolyte gel was prepared by mixing 1 g PVA and 0.5 g NaCl in 10 mL deionized water and stirring for 12 hours at room temperature. Patternable solid-state PVA-based electrolyte was synthesized by mixing 1 g poly (vinyl alcohol) (PVA, from Sigma Aldrich) and 5 mg potassium dichromate ($K_2Cr_2O_7$, as photoinitiator) in 10 ml DI Water at 80°C for 12 hours. 0.5 g NaCl was added to the above solution and then spined coated into ~2 μm PVA films. These PVA films were then photo-exposed (365 nm, 15 W cm^{-2}) for 90 s through a photomask and patterned by developing in DI water for 20 s.

Patternable solid-state PAA-based electrolyte was synthesized by adding 0.18 g lithium chloride (LiCl) into 5 mL deionized water, which was heated to 90°C. Then 0.6 g gelatin was added to above solution to get yellow clear solution and cooled to room temperature to form solution A. Meanwhile, 3 mL acrylic acid (AA) was naturalized by 0.37 g sodium hydroxide (NaOH), forming Solution B. Finally, Solution B was added

dropwise to Solution A, followed by the addition of 0.05 g of N,N'-Methylenebis(acrylamide) (MBAA) and 0.1 g of 2-Hydroxy-4'-(2-hydroxyethoxy)-2-methylpropiophenone (photoinitiator 2959). The synthesized electrolyte was subsequently patterned into desired configurations using spin-coating and photolithography processes.

Supplementary Note 3: Theory of DRT analysis

Distribution of Relaxation Times (DRT) is a powerful analytical method used in materials science, especially in the study of electrochemical systems such as ion batteries (*38, 39*). This work marks the first application of DRT to organic electrochemical transistors.

Concept: The DRT approach assumes that the system's overall response is a superposition of multiple relaxation processes, each with its own relaxation time τ. The distribution of these relaxation times gives insight into the various underlying processes, as follows (*24*):

1. Bulk conductivity (Ohmic losses): nanoseconds to milliseconds;
2. Charge transfer (electrochemical reaction): milliseconds to seconds;
3. Solid diffusion (Warburg impedance): seconds to hours.

Mathematical Representation: The target of the DRT is to transform the timescale characteristics of frequency-based EIS in the time domain. It is an effective assumption to simulate the convergent impedance of the electrochemistry system as the series connection of Ohm impedance (R_0) with polarization impedance (R_{pol}) induced by electrochemical processes. The DRT approach underpins the determination of a timescale (τ) distribution, which is obtained by solving the following Fredholm integral equation for the latent distribution $\gamma(\tau)$ (*40*):

$$Z_{DRT}(f) = R_0 + R_{pol} \int_0^\infty \frac{\gamma(\tau)}{1 + i\pi f \tau} d\tau \qquad (S1)$$

where $Z_{DRT}(f)$ is the impedance obtained with the DRT model and f is the frequency. DRT from the EIS data were calculated by MatlabR2023b with a toolbox of DRT-TOOLS developed by the research group of Professor Francesco Ciucci, available at https://github.com/ciuccislab/ DRT-Survey.

Supplementary Note 4: Evidences that that polycations in pDADMAC are mobile

To determine if the ions in pDADMAC are mobile, the pDADMAC solution was dropped onto the PEDOT:PSS film while applying a voltage (Fig. S8). The result reveals that both anions (Fig. S9 and Supplementary Video 2) and cations (Fig. 2H and Supplementary Video 3) are able to migrate under the electric field. Moreover, during measurements of the liquid-state vertical OECT array (Fig. S10), small droplets of pDADMAC were observed to move only along the charged drain line (V_D = -0.5 V, left in Fig 2I) from the electrolyte edge, but the uncharged line remained clean (right in Fig 2I). This phenomenon further provides solid evidence that the polycations in pDADMAC are indeed mobile. Since pDADMAC polyelectrolytes can move laterally at the micrometer or even millimeter scale in a short time, vertical movement at the nanometer scale is also feasible (*41*), which is supported by the bulk electrochromic behaviour.

Generally, PEDOT:PSS-based OECTs operate in depletion mode, where the negatively charged PSS^- counterions are balanced by cations (M^+) from the electrolyte driven by a positive gate voltage (*42, 43*). This interaction facilitates the reduction of $PEDOT^+$ cations (i.e., de-doping), causing a decrease in the number of free charge carriers (holes) in PEDOT:PSS and, consequently, a reduction in the channel's electronic conductivity and the color change of the PEDOT:PSS channel, as described by the following reaction:

$$PEDOT^+: PSS^-(\textbf{dark blue}) + M^+ + e^- \Leftrightarrow PEDOT^0(\textbf{light blue}) + M^+: PSS^- \quad (S2)$$

Supplementary Note 5: Influence of gate materials

Besides structural modulation, the nature of gate electrodes also affects the ion transport efficiency because it determines the voltage drop and capacitance across the gate-electrolyte interface, ultimately modulating the electrochemical process(*3*). As shown in Fig. S14, Ag, Au, and PEDOT:PSS are patterned as the gate electrodes in the top frame drain-based vertical OECTs, each requiring 1.3 V, 2 V, and 3 V, respectively, to produce similar changes in PSC. In fact, the corresponding threshold voltages of bottom frame drain-based vertical OECTs are 0.8 V, 1.6 V, and 2.0 V (Fig. 5C), respectively. Here, higher presynaptic voltages are needed to push the polycations into the channels beneath the top drains. And the spike gate voltages can prevent the damage caused by a continuous sweep of V_G at the same magnitude (Fig. 1J (ii)) owning to the relaxation periods provided by the spikes. On the other hand, gates based on Ag and PEDOT:PSS exhibit faster modulation compared to those based on Au gates (Fig. 3G).

Supplementary Note 6: Dimension-dependent investigation

The statistics shown in Fig. 5I (i) and (ii) reveal that variations in l_{FO} and l_{PP} have no substantial effect on the on/off ratio, I_{on}, and g_m. However, an increase in the inner size of the bottom frame significantly reduces I_{on} and g_m, as shown in Fig. 5I (iii). On the contrary, these two performance metrics exhibit an upward trend with increasing the PEDOT:PSS thickness (h_{PP}, Fig. 5I (iv)) and hole size in the passivation layer (l_{PH}, Fig. 5I (v)). These phenomena can be explained by the simulated electric field distributions, as shown in Fig. 5J. Fig. 5J (i) and (ii) suggest that the outer size of the bottom frame (l_{FO}) has a negligible impact on the vertical electric field, consistent with the tendency observed in Fig. 5I (i). On the other hand, shrinking the inner size of the bottom frame (l_{FI}) can reduce the lateral electric field path and increase the vertical proportion (Fig. 5J (i) and (iii)), resulting in increased I_{on} and g_m (Fig. 5I (iii)). Similarly, the improvement induced by enlarging the passivation hole, as depicted in Fig. 5I (iv), is attributed to the reduction in the lateral electric field proportion (Fig. 5J (iii) and (iv)). The rise in I_{on} and g_m, along with a slight increase in the on/off ratio shown in Fig. 5I (v) can be associated with the increased conductivity of the channel due to the increase in the PEDOT:PSS thickness.

Supplementary Figures

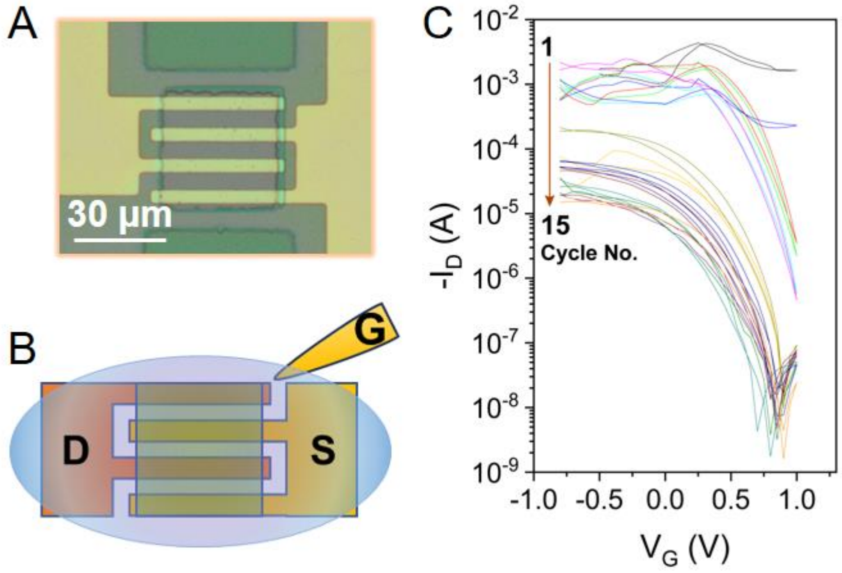

Fig. S1. Stabilization of lateral OECT based on liquid-state pDADMAC electrolyte. (**A** and **B**) Optical and schematic images of the liquid-state lateral OECTs. (**C**) Stabilization process of the lateral OECT based on liquid-state pDADMAC electrolyte.

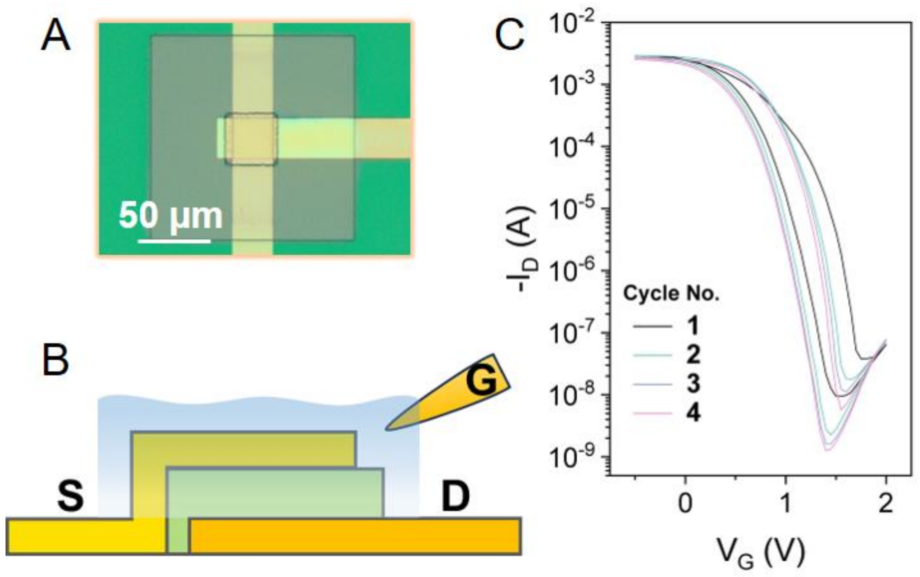

Fig. S2. Stabilization of vertical OECT based on liquid-state PBS electrolyte. (**A** and **B**) Optical and schematic images of the liquid-state vertical OECTs. (**C**) Stabilization process of the vertical OECT based on liquid-state PBS electrolyte.

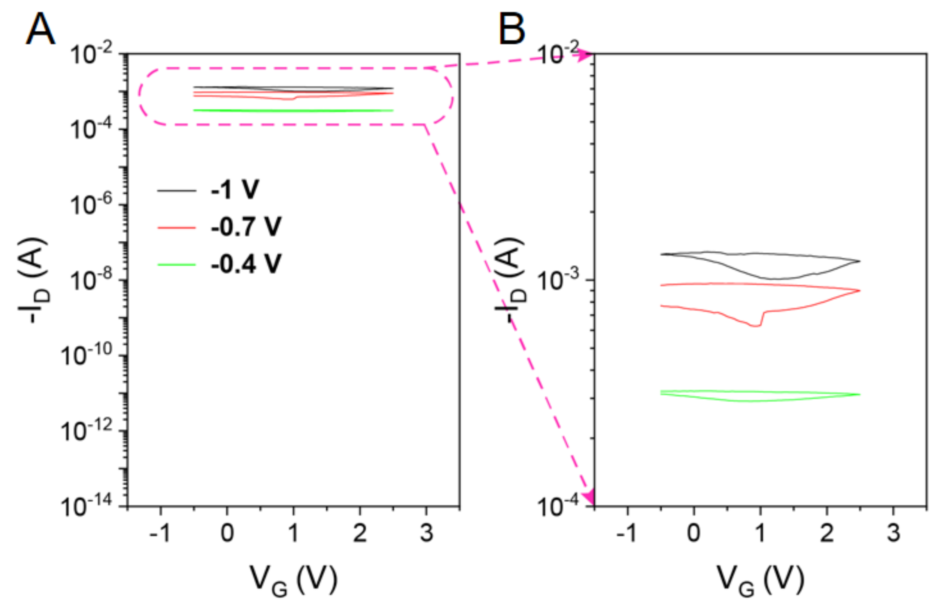

Fig. S3. Initiation failure of pDADMAC-based solid-state vertical OECTEM at lower gate voltage. (**A** and **B**) The I_D v.s. V_G curves and the enlarged image.

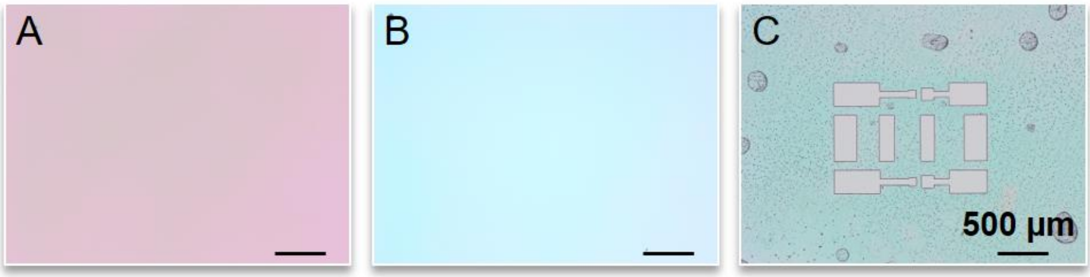

Fig. S4. Contamination from photolithography process. (**A**) PEDOT:PSS film on silicon wafer. (**B** and **C**) AZ 5214E photoresist on PEDOT:PSS before (**B**) and after (**C**) development.

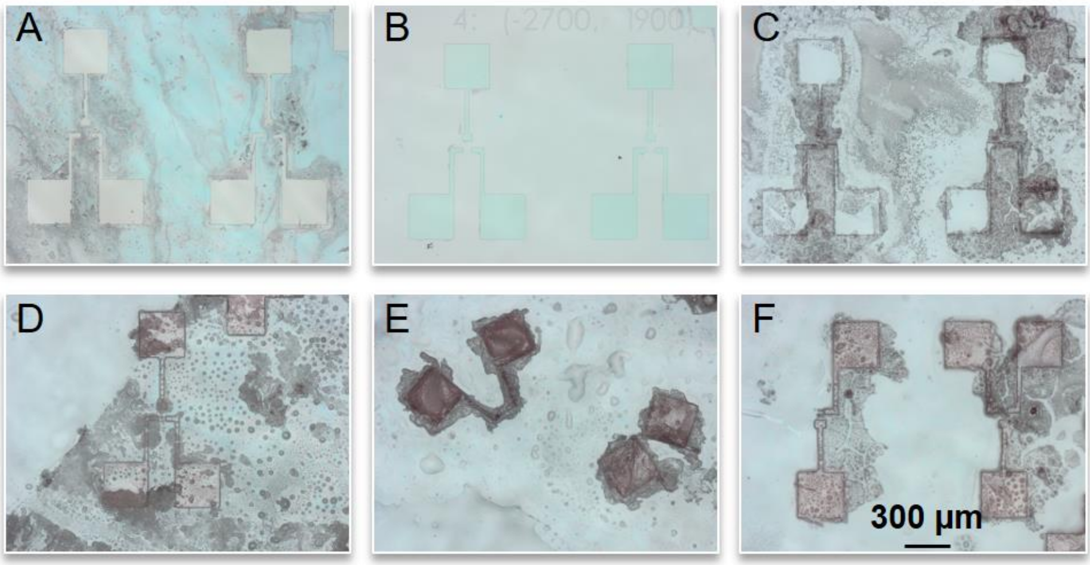

Fig. S5. Various failure in patterning pDADMAC. All images are at the same magnification and share the same scale bar.

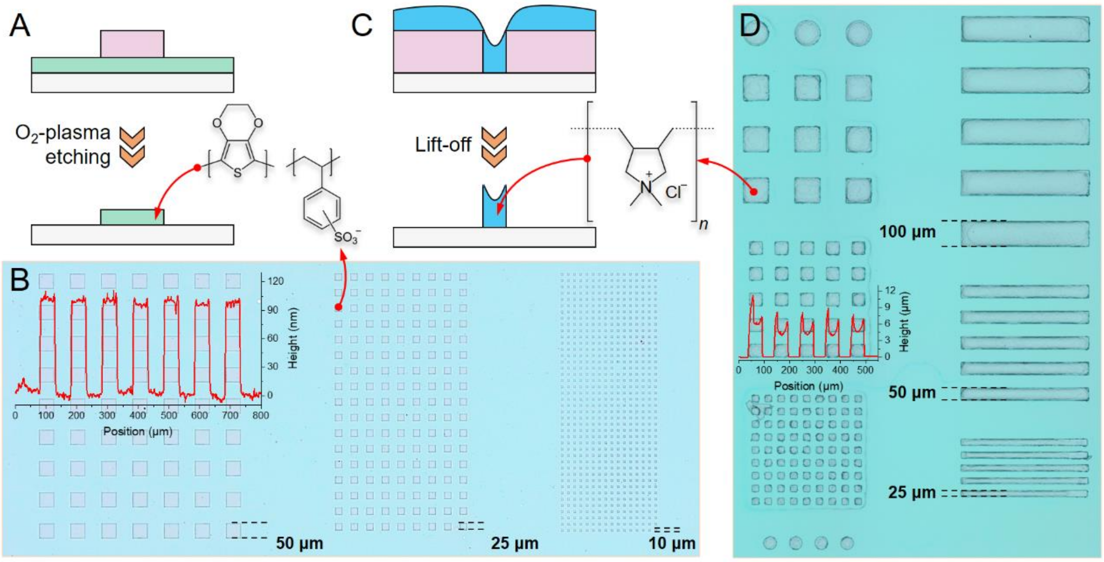

Fig. S6. Height profiles of PDEOT:PSS and pDADMAC patterns. (**A** and **B**) The pattern process and the corresponding height profile of PEDOT:PSS patterns. (**C** and **C**) The pattern process and the corresponding height profile of pDADMAC patterns.

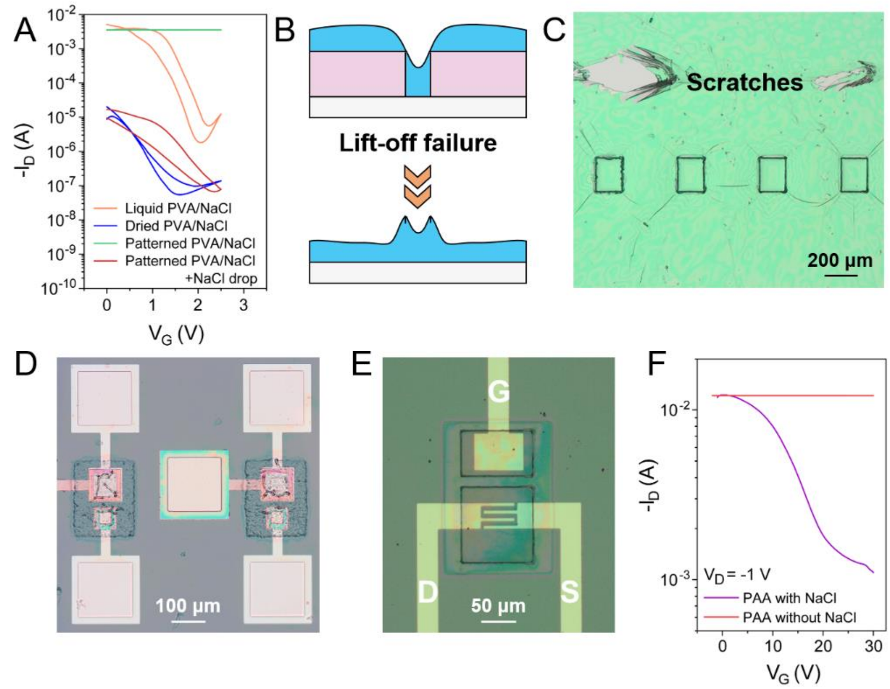

Fig. S7. Patterned solid-state electrolytes. (**A**) Representative transfer characteristics of liquid- and solid-state PVA electrolytes. (**B** and **C**) The lift-off process developed for pDADMAC patterns is inefficient for PVA gel, as the PVA remains intact as a whole film after photoresist removal. (**D**) Optical photo of OECTs based on photolithographically patterned PVA electrolyte. (**E** and **F**) Representative transfer characteristics and optical photo of OECTs based on photolithographically patterned PAA electrolyte.

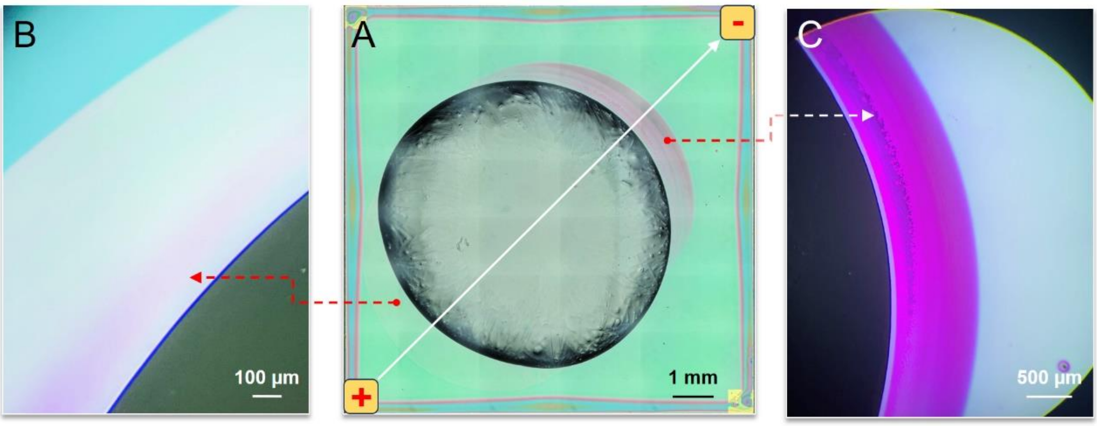

Fig. S8. Movement test of pDADMAC under electric field. (**A**) The overall movement of the pDADMAC droplet onto PEDOT:PSS film under 10 V. (**B** and **C**), The movements of anions (**B**) and cations (**C**) under the electric field.

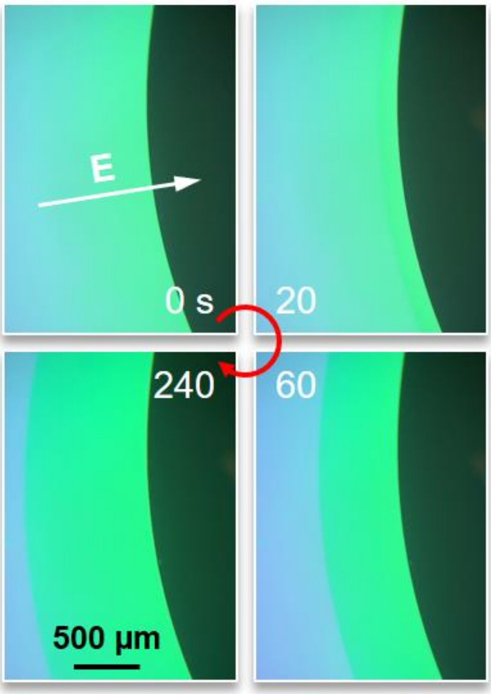

Fig. S9. Movement process of anions in pDADMAC. All images are at the same magnification and share the same scale bar. Extracted from Supplementary Video 2.

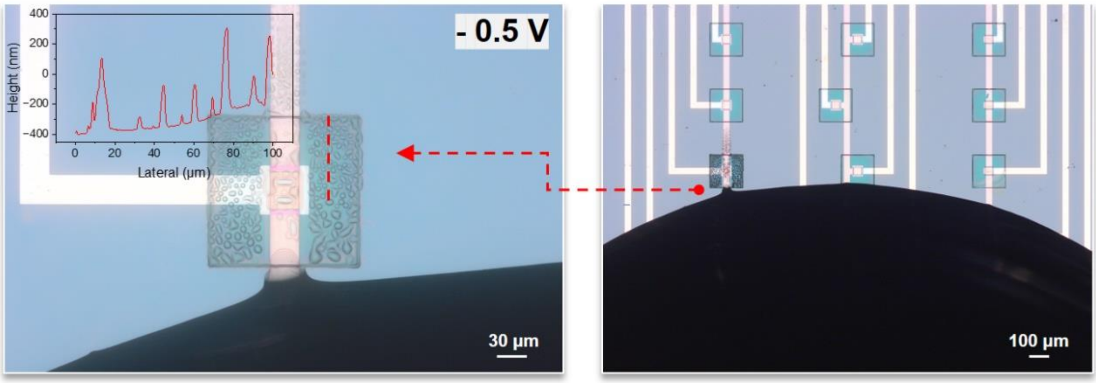

Fig. S10. Liquid-state vertical OECT array under test. The height profile demonstrates the driven pDADMAC small segments along the charged drain line (V_D = -0.5 V).

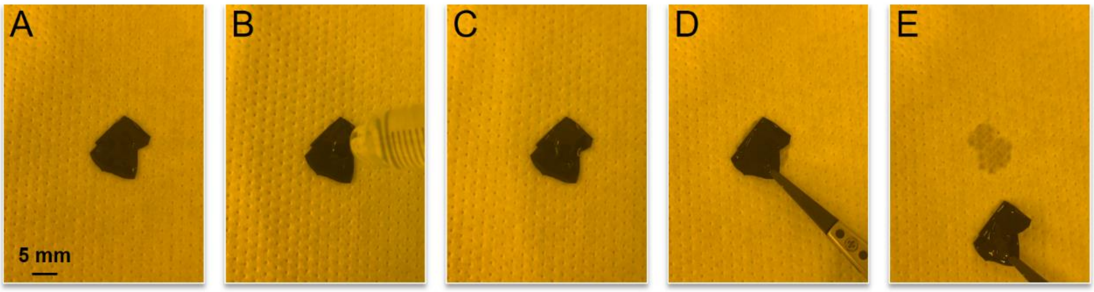

Fig. S11. Penetration of pDADMAC after dropping onto the PEDOT:PSS thick film. (**A**) Freestanding thick PEDOT:PSS sheets. (**B**) Dropping liquid pDADMAC onto PEDOT:PSS sheets. (**C**) Penetrating. (**D**) Removing the PEDOT:PSS sheets. (**E**) Soaked paper. All images are at the same magnification and share the same scale bar. Extracted from Supplementary Video 4.

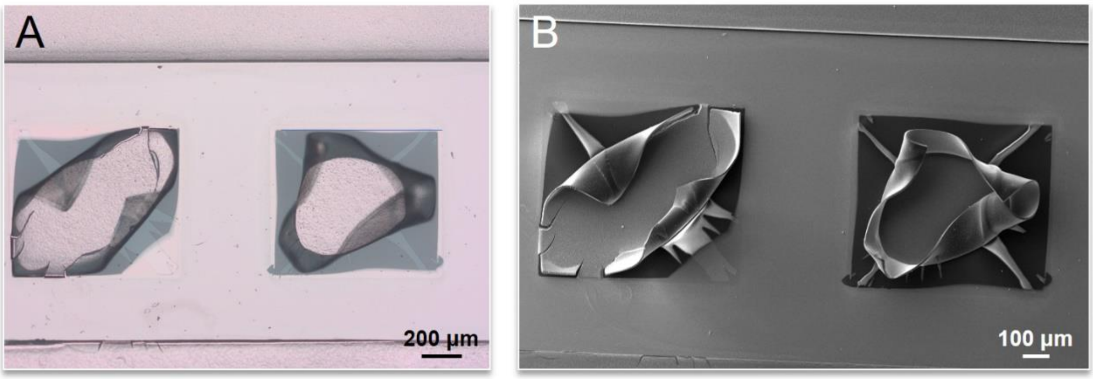

Fig. S12. Delamination of the pDADMAC patterns in vacuum directly tore the underlying PEDOT:PSS film. (**A**) Peeling in e-beam chamber. (**B**) Further peeling in SEM chamber.

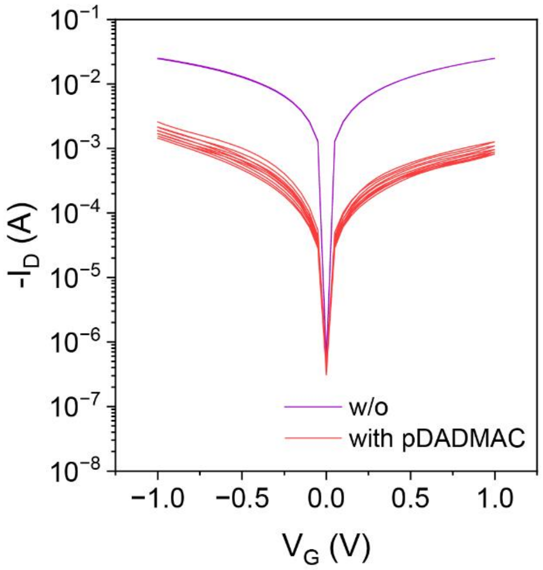

Fig. S13. Decrease in I_D current after the patterning of pDADMAC. PEDOT:PSS thickness: ~30 nm. V_D = -0.5 V.

Compared to a bare PEDOT:PSS film, the conductivity of the solid-state pDADMAC-covered PEDOT:PSS film decreased (Fig. S13), possibly because the electrostatic interaction between pDADMAC and PSS could disrupt the efficient transfer of holes among PEDOT chains within the PSS matrix and partially de-dope the PEDOT component.

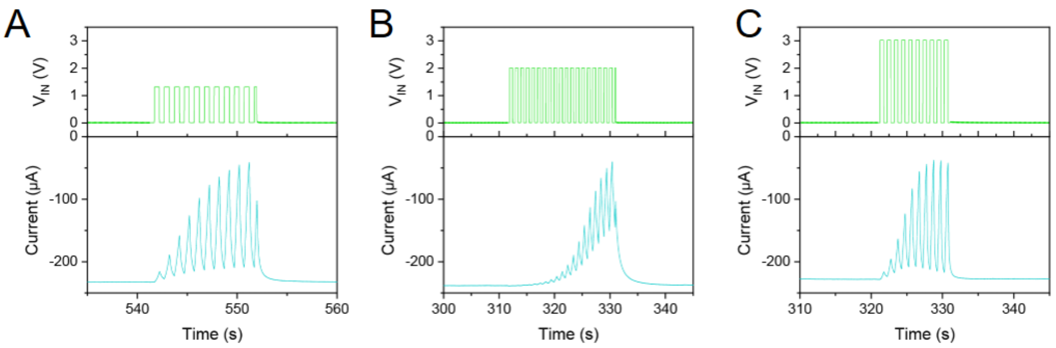

Fig. S14. Performance based Ag, Au, and PEDOT:PSS gate electrodes. (**A**) Based on Ag gate. (**B**) Based on Au gate. (**C**) Based on PEDOT:PSS gate. Frequency = 1 Hz, V_D = -0.2 V.

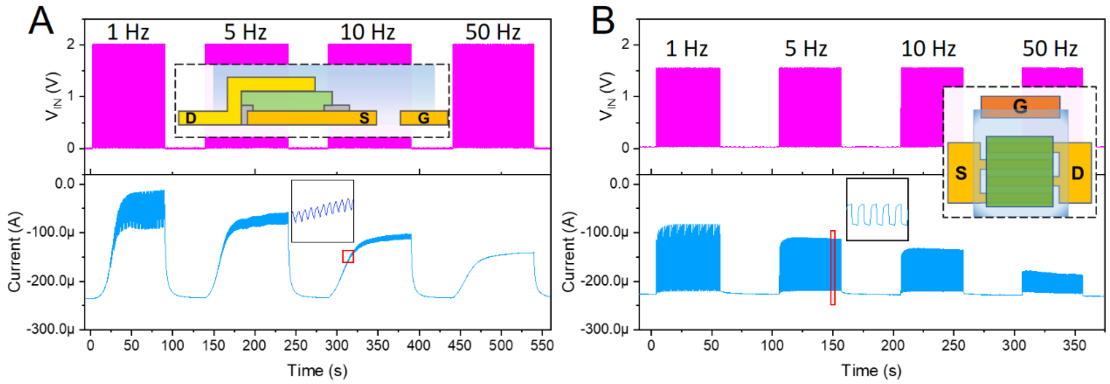

Fig. S15. Spike frequency-dependent modulation. (**A** and **B**) Spike frequency-dependent modulations of top planar drain-based vertical OECT (**A**) and lateral OECT (**B**). V_D = -0.2 V.

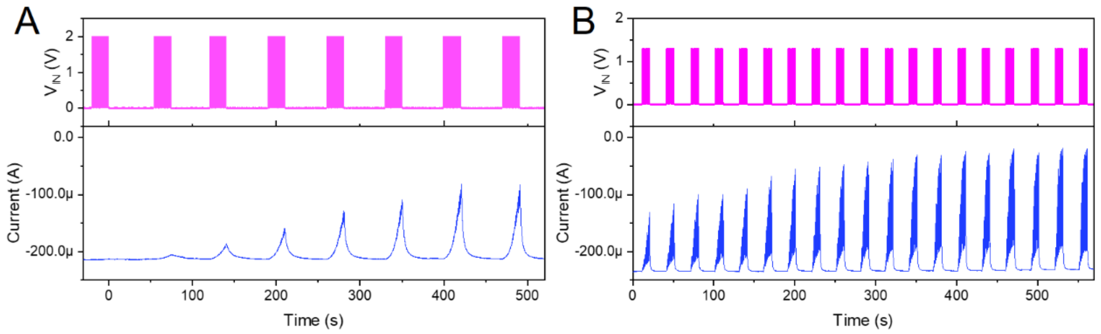

Fig. S16. Time interval-dependent modulation. (**A**) Vertical OECTs based on top planar drains and Au gate. (**B**) Vertical OECTs based on top frame drains and Ag gate. Frequency = 1 Hz, V_D = -0.2 V.

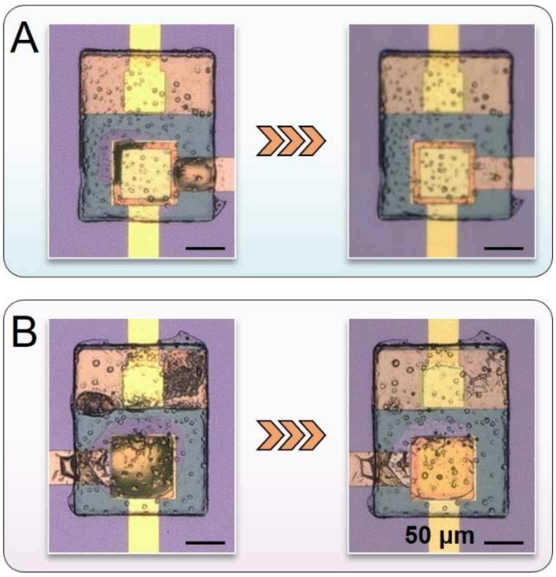

Fig. S17. Morphological changes observed in TF-*v*OECTs. (**A**) Middel V_G (e.g., 3 V) leads to reversible morphology change. (**B**) High V_G (e.g., 5 V) leads to irreversible morphology change. It is worth noting that the morphology changes are reversible by properly adjusting the gate voltages; otherwise, the device will be destroyed due to excessive deformation of the top drains.

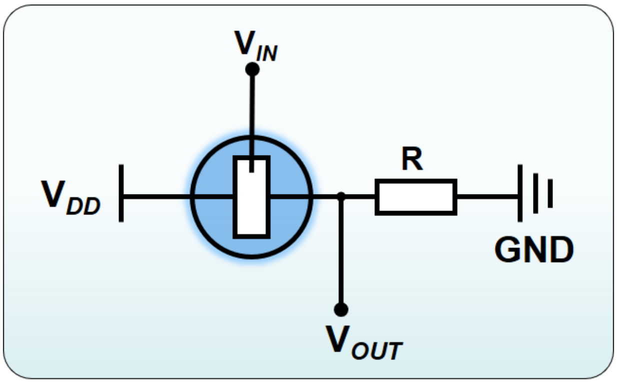

Fig. S18. A standard inverter circuit where a resistor (830 Ω here) is placed in series with the OECT.

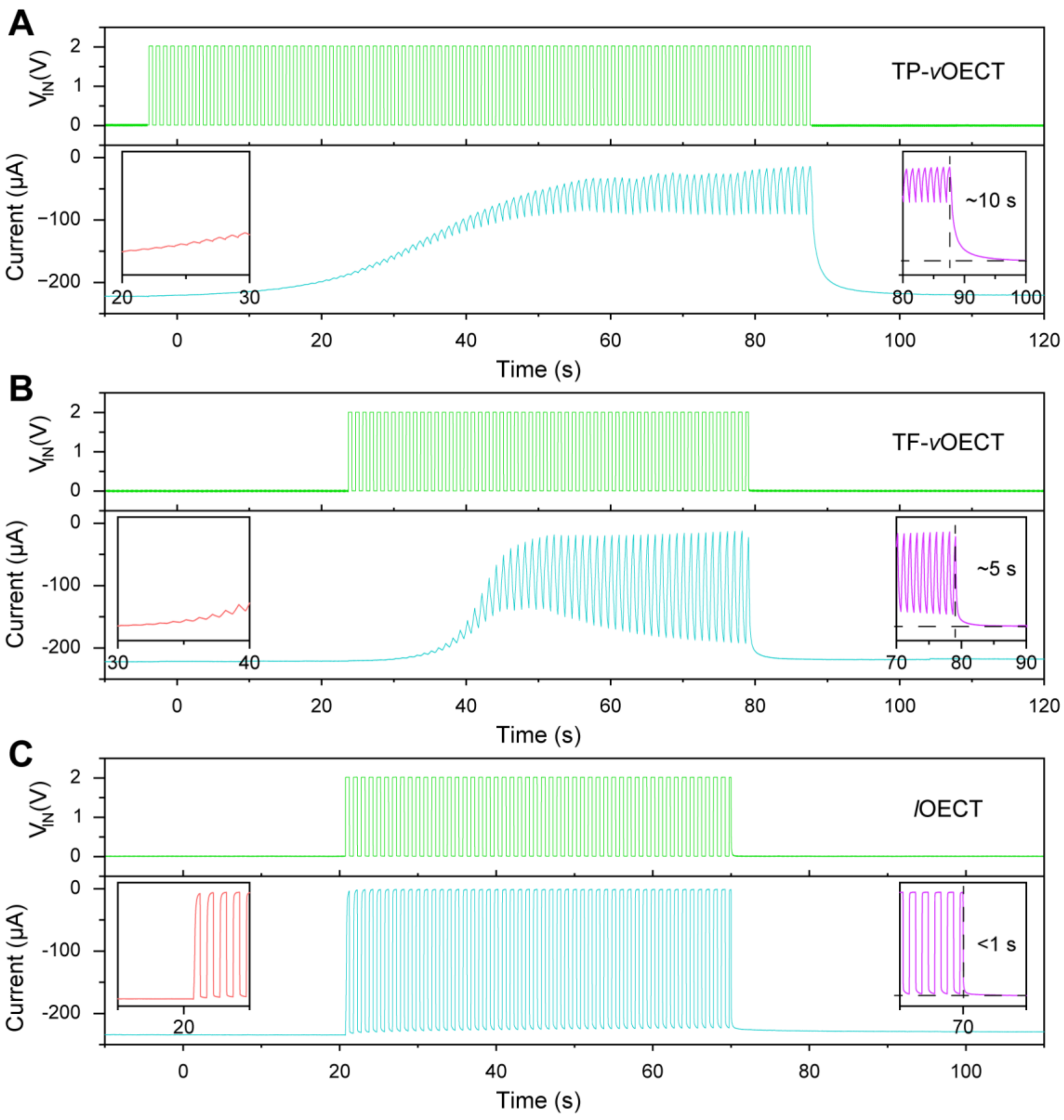

Fig. S19. Enlarged versions of Fig. 3B-D presented in manuscript. Plasticity performances of (**A**) TP-*v*OECTs, (**B**) TF-*v*OECTs and (**C**) *l*OECTs. Upon applying multiple spikes (1 Hz), the PSCs are progressively inhibited step by step, resulting in distinct conductance states (see insets).

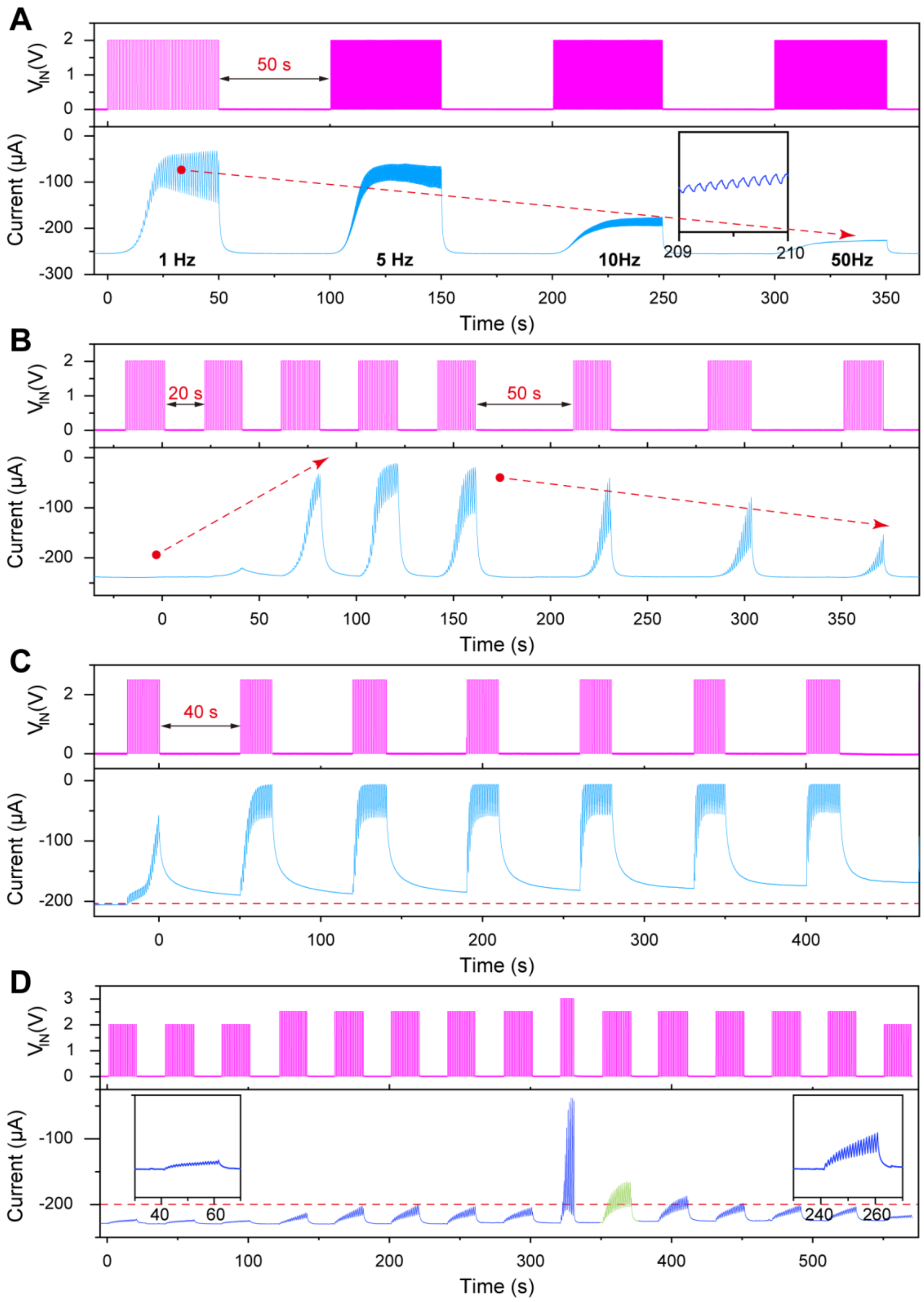

Fig. S20. Enlarged versions of Fig. 3H, I, J and L presented in manuscript. (**A**) Frequency-dependent plasticity of TF-*v*OECTs with spike (0→2 V) frequencies ranging from 1, 5, 10, and 50 Hz. (**B**) Short-term depression (STD)-based long-term memory (short time interval, **left**) and forget (long time interval, **right**) at 1 Hz. (**C**) Long-term depression (LTD) triggered by high presynaptic voltages at 1 Hz. (**D**) Voltage-dependent training-learning behaviour of TF-*v*OECTs based on PEDOT:PSS gate at 1 Hz.

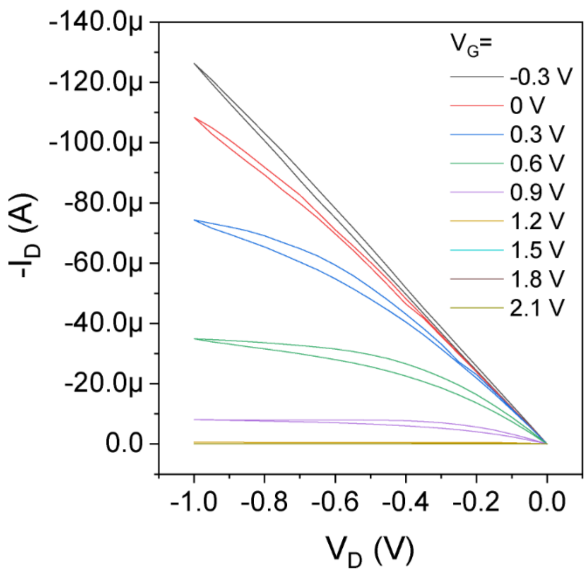

Fig. S21. Transfer curves of bottom frame drain-based solid-state vertical OECTs.

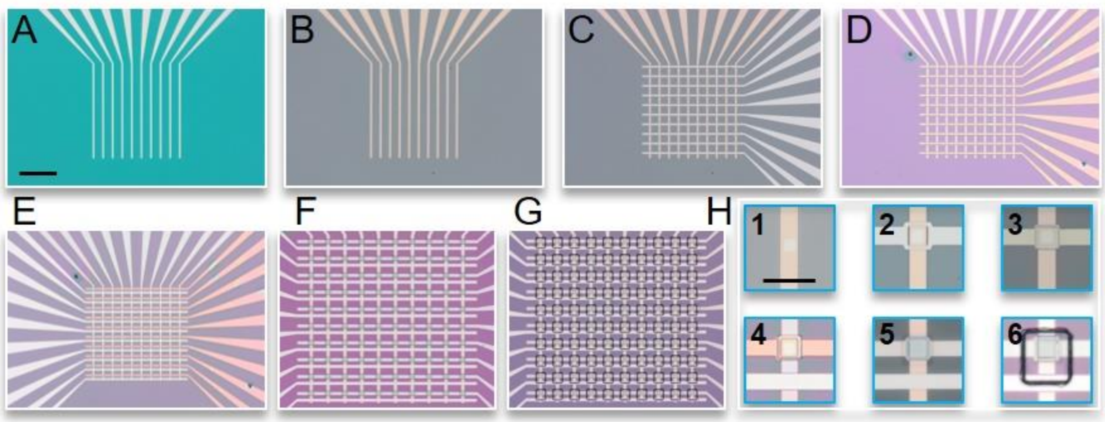

Fig. S22. Fabrication process of OECT array. (**A**) Bottom source lines. (**B**) First passivation with ALD-Al_2O_3 (10 nm). (**C**) Bottom drain lines. (**D**) Second passivation with SU-8 (300 nm). (**E**) Gate lines. (**F**) PEDOT:PSS patterns. (**G**) pDADMAC patterns. (**H**) Enlarge images of (**B-H**). Figures in (**A-G**) are at the same magnification and share the same scale bar (100 μm). Figures in (**H)** are at the same magnification and share the same scale bar (50 μm).

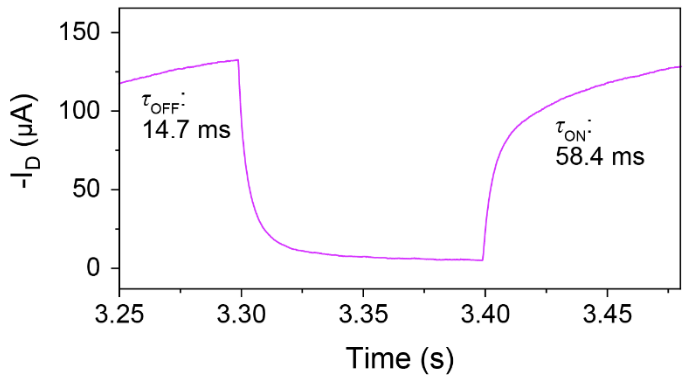

Fig. S23. Transient response of lateral OECT.

The turn-on and turn-off transient times (τ_{ON} and τ_{OFF}) are a few milliseconds (Fig. 4m), shorter than those of their lateral counterparts (Fig. S21). Huang et al. (*5*) reported longer τ_{ON} and τ_{OFF} for top planer Au drain-based vertical OECTs than those of the corresponding lateral OECTs using PBS solution, due to ion transport delays caused by the solid top electrode. However, in our bottom frame-based vertical OECT, the fully exposed PEDOT:PSS to the electrolyte and a more directly oriented gating electric field towards the channel result in shorter switching times and reduced hysteresis compared to the lateral OECT.

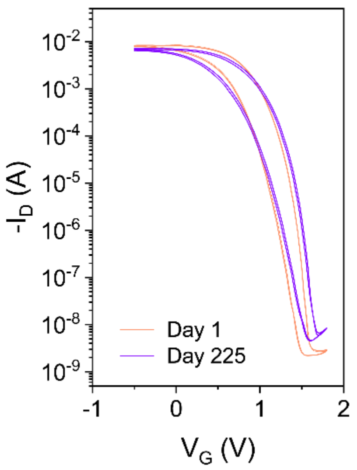

Fig. S24. BF-vOECT's durability and reliability under ambient condition over 225 days.

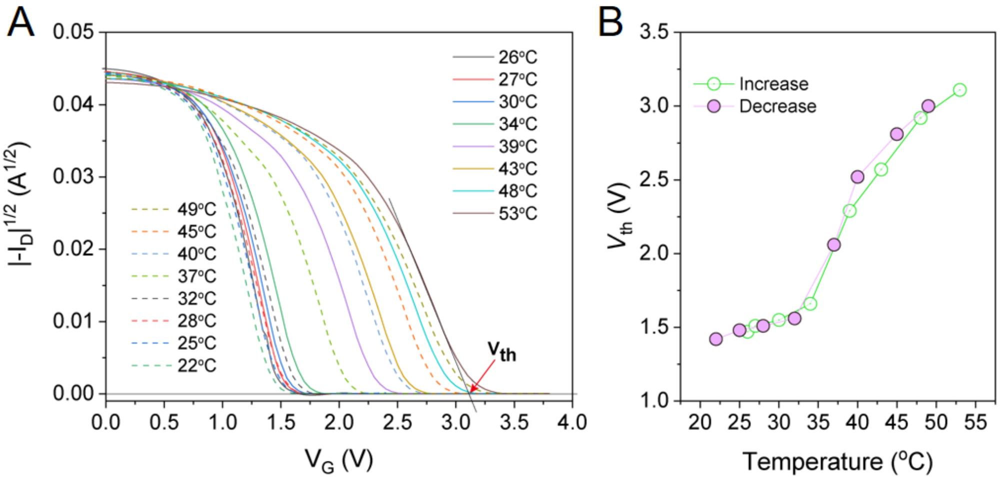

Fig. S25. Temperature-dependent threshold voltage. (**A**) Extraction of threshold voltages at different temperature. (**B**) The extracted threshold voltages.

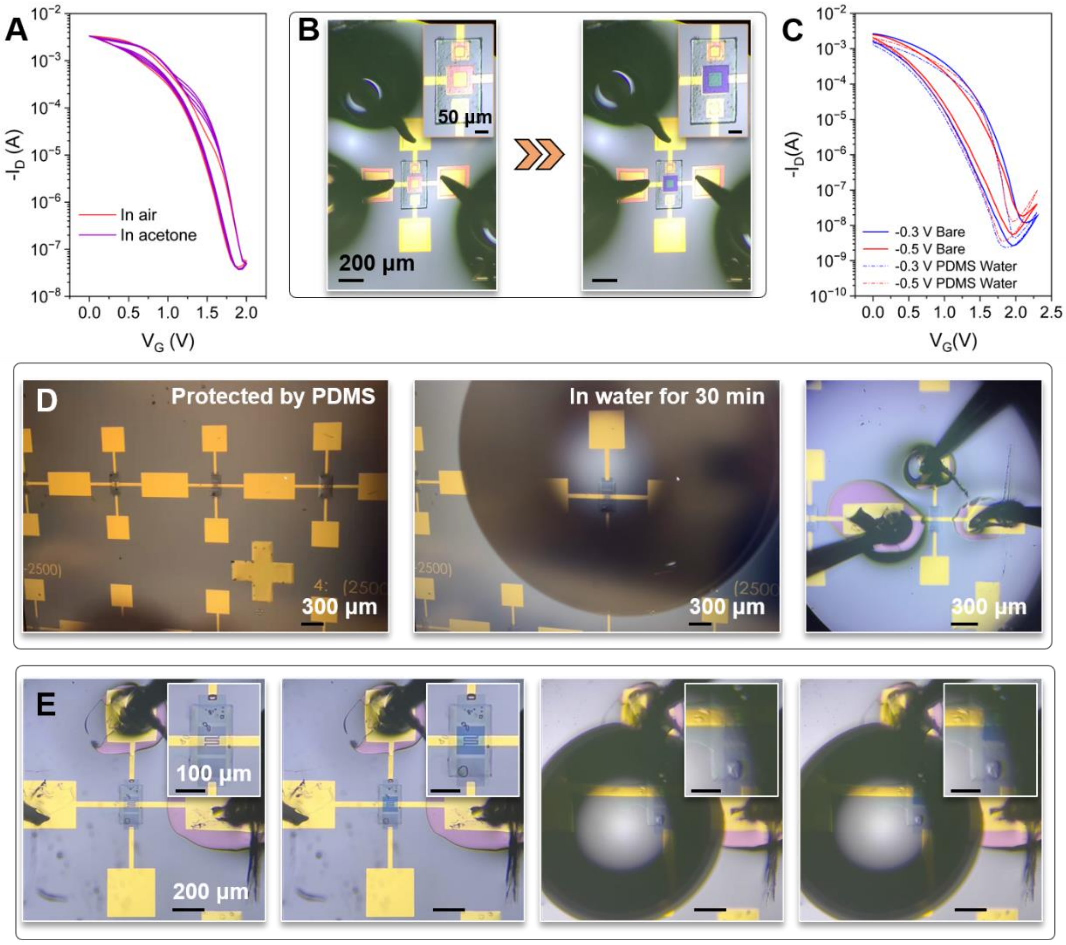

Fig. S26. Stability in solutions. (**A**) Transfer characteristics of bottom frame drain-based vertical OECTs in air and acetone. (**B**) Electrochromic performance of bottom frame drain-based vertical OECTs in acetone. Extracted from Supplementary Video 5. (**C** and **D**) Representative transfer characteristics and working stability in water after being sealed with PDMS. (**E**) Electrochromic behavior in water after being sealed with PDMS. Extracted from Supplementary Video 6.

To prevent the non-crosslinked pDADMAC patterns from dissolving in water, a layer of PDMS was spin-coated on top to seal the OECTs. As illustrated in Fig. S26C and D, the devices exhibit negligible performance degradation before and after exposure to water. Additionally, the electrochromic behavior of the OECTs remains clearly observable in water (Fig. S26E), successfully extending the pDADMAC-based solid-state OECTs to aqueous environments.

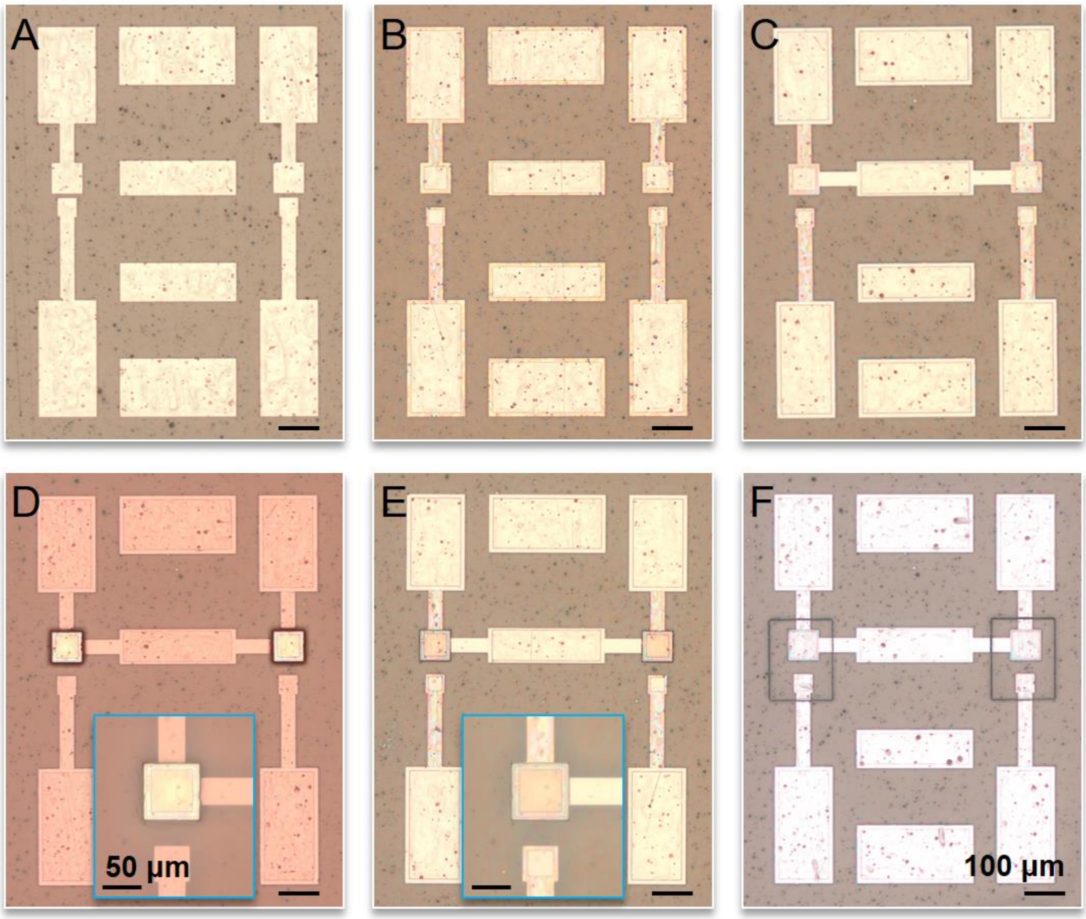

Fig. S27. Fabrication of flexible OECTs. (**A**) Bottom source and gate. (**B**) Passivation with SU-8. (**C**) Bottom frame drain. (**D**) Spin-coating PEDOT:PSS film onto AR-P 5910 patterns. (**E**) Lift-off of PEDOT:PSS film. (**F**) pDADMAC patterns. All figures (except the insets) are at the same magnification and share the same scale bar.

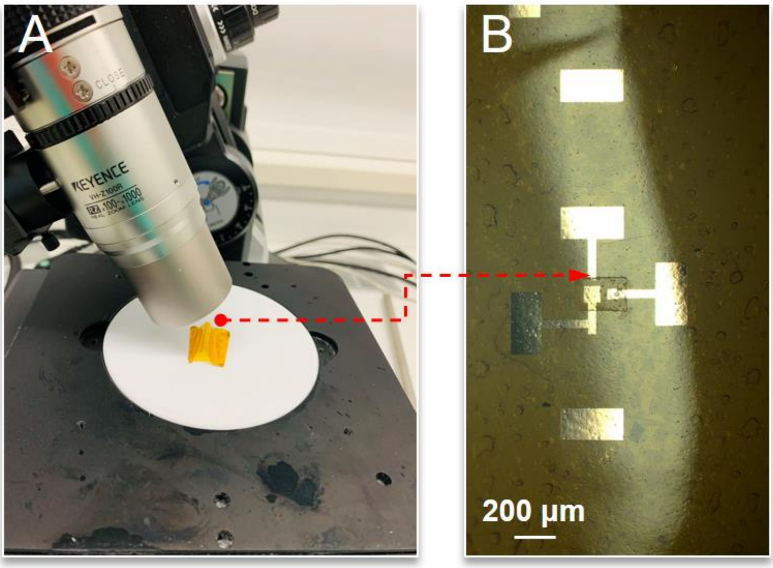

Fig. S28. Bending of flexible OECTs. (**A**) The bent flexible OECTs. (**B**) Optical image of the bent OECTs.

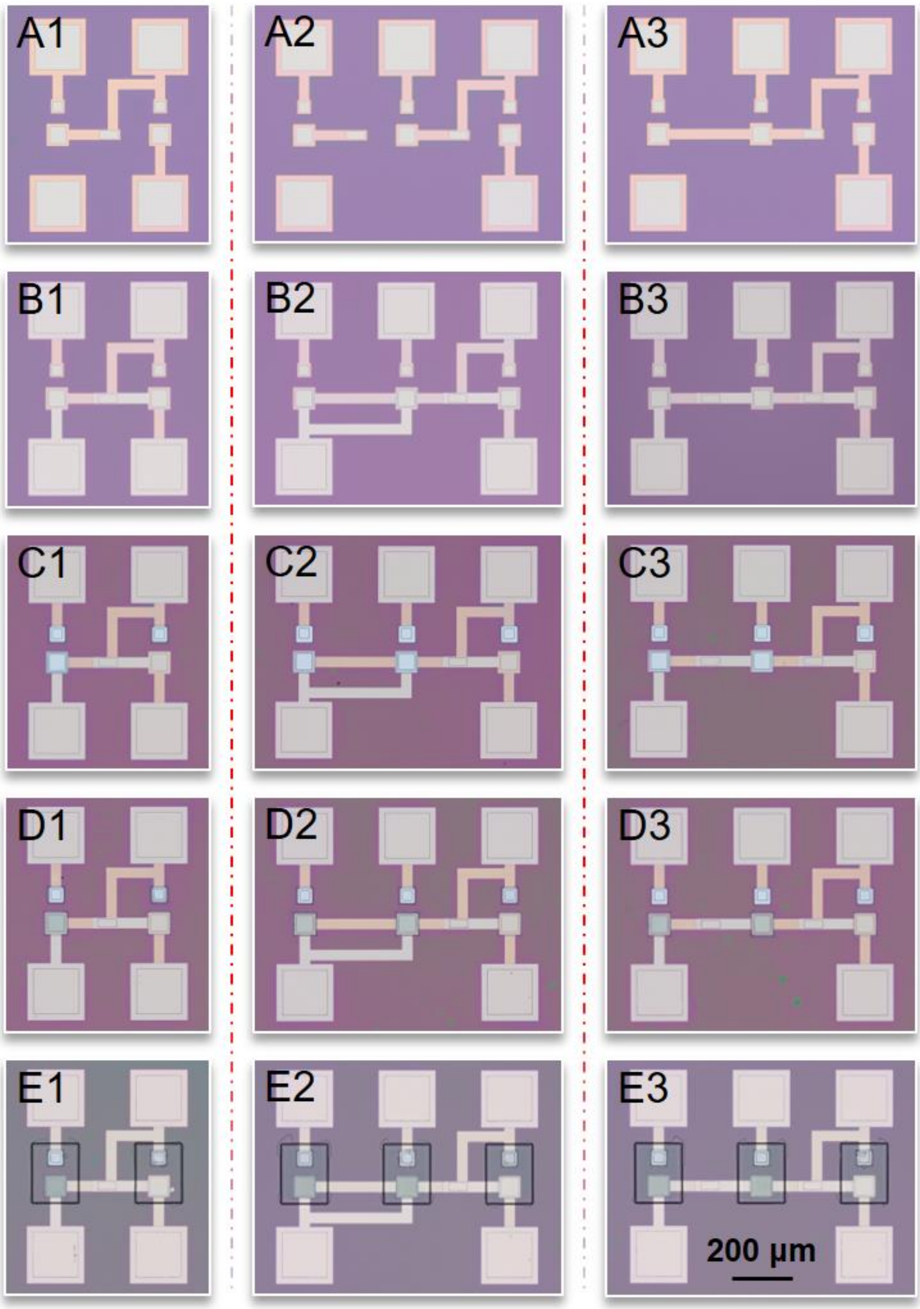

Fig. S29. Fabrication of NOT (left), NAND (middle), and NOR (right) gates. (**A**) Passivation with SU-8. (**B**) Bottom frame drain. (**C**) Thick PEDOT:PSS patterns via dry etching. (**D**) Thin PEDOT:PSS patterns via lift-off. (**E**) pDADMAC patterns via lift-off. All figures are at the same magnification and share the same scale bar.

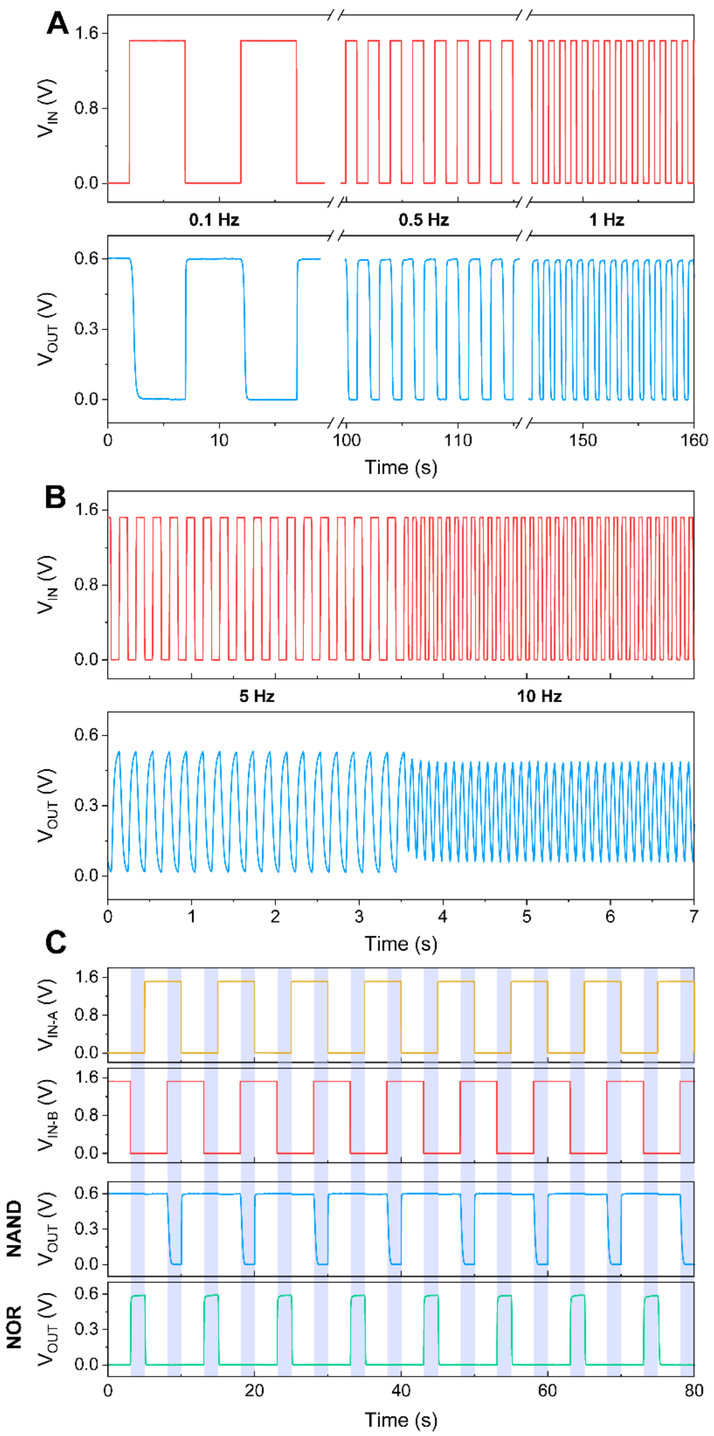

Fig. S30. Transient measurement of the Boolean inverter, NAND and NOR. (**A** and **B**) Switching performance of the inverter. (**C**) Switching performances of the NAND and NOR.

Captions for the Supplementary Videos

Supplementary Video 1: Electrochromic performance of solid-state lateral OECT. Speed: 1X.

Supplementary Video 2: Drift of anions under electric field. Speed: 10X.

Supplementary Video 3: Drift of cations under electric field. Speed: 10X.

Supplementary Video 4: Penetration of pDADMAC into PEDOTPSS sheet. Speed: 1X.

Supplementary Video 5: Reliable operation and electrochromic performance in acetone. Speed: 4X.

Supplementary Video 6: Reliable operation and electrochromic performance in water after PDMS encapsulation. Speed: 2X.

Supplementary Video 7: Electrophysiological signal recording from *Mimosa pudica*. Speed: 1X.

REFERENCES AND NOTES

1. F. Torricelli, D. Z. Adrahtas, Z. Bao, M. Berggren, F. Biscarini, A. Bonfiglio, C. A. Bortolotti, C. D. Frisbie, E. Macchia, G. G. Malliaras, I. M. Culloch, M. Moser, T.-Q. Nguyen, R. M. Owens, A. Salleo, A. Spanu, L. Torsi, Electrolyte-gated transistors for enhanced performance bioelectronics. *Nat. Rev. Methods Primers* **1**, 66 (2021).

2. J. Rivnay, S. Inal, A. Salleo, R. M. Owens, M. Berggren, G. G. Malliaras, Organic electrochemical transistors. *Nat. Rev. Mater.* **3**, 17086 (2018).

3. D. Ohayon, V. Druet, S. Ina, A guide for the characterization of organic electrochemical transistors and channel materials. *Chem. Soc. Rev.* **52**, 1001–1023 (2023).

4. B. D. Paulsen, K. Tybrandt, E. Stavrinidou, J. Rivnay, Organic mixed ionic-electronic conductors. *Nat. Mater.* **19**, 13–26 (2020).

5. P. Andersson Ersman, R. Lassnig, J. Strandberg, D. Tu, V. Keshmiri, R. Forchheimer, S. Fabiano, G. Gustafsson, M. Berggren, All-printed large-scale integrated circuits based on organic electrochemical transistors. *Nat. Commun.* **10**, 5053 (2019).

6. K. Guo, S. Wustoni, A. Koklu, E. Diaz-Galicia, M. Moser, A. Hama, A. A. Alqahtani, A. N. Ahmad, F. S. Alhamlan, M. Shuaib, A. Pain, I. McCulloch, S. T. Arold, R. Grunberg, S. Inal, Rapid single-molecule detection of COVID-19 and MERS antigens via nanobody-functionalized organic electrochemical transistors. *Nat. Biomed. Eng.* **5**, 666–677 (2021).

7. W. Lee, S. Kobayashi, M. Nagase, Y. Jimbo, I. Saito, Y. Inoue, T. Yambe, M. Sekino, G. G. Malliaras, T. Yokota, M. Tanaka, T. Someya, Nonthrombogenic, stretchable, active multielectrode array for electroanatomical mapping. *Sci. Adv.* **4**, eaau2426 (2018).

8. X. Ji, B. D. Paulsen, G. K. K. Chik, R. Wu, Y. Yin, P. K. L. Chan, J. Rivnay, Mimicking associative learning using an ion-trapping non-volatile synaptic organic electrochemical transistor. *Nat. Commun.* **12**, 2480 (2021).

9. C. Jiang, J. Liu, Y. Ni, S. Qu, L. Liu, Y. Li, L. Yang, W. Xu, Mammalian-brain-inspired neuromorphic motion-cognition nerve achieves cross-modal perceptual enhancement. *Nat. Commun.* **14**, 1344 (2023).

10. R. Shameem, L. M. Bongartz, A. Weissbach, H. Kleemann, K. Leo, Hysteresis in organic electrochemical transistors: relation to the electrochemical properties of the semiconductor. *Appl. Sci.* **13**, 5754 (2023).

11. G. D. Spyropoulos, J. N. Gelinas, D. Khodagholy, Internal ion-gated organic electrochemical transistor: A building block for integrated bioelectronics. *Sci. Adv.* **5**, eaau7378 (2019).

12. S. Wang, X. Chen, C. Zhao, Y. Kong, B. Lin, Y. Wu, Z. Bi, Z. Xuan, T. Li, Y. Li, W. Zhang, E. Ma, Z. Wang, W. Ma, An organic electrochemical transistor for multi-modal sensing, memory and processing. *Nat. Electron.* **6**, 281–291 (2023).

13. Y. Choi, D. H. Ho, S. Kim, Y. J. Choi, D. G. Roe, I. C. Kwak, J. Min, H. Han, W. Gao, J. H. Cho, Physically defined long-term and short-term synapses for the development of reconfigurable analog-type operators capable of performing health care tasks. *Sci. Adv.* **9**, eadg5946 (2023).

14. J. Kim, R. M. Pankow, Y. Cho, I. D. Duplessis, F. Qin, D. Meli, R. Daso, D. Zheng, W. Huang, J. Rivnay, T. J. Marks, A. Facchetti, Monolithically integrated high-density vertical organic electrochemical transistor arrays and complementary circuits. *Nat. Electron.* **7**, 234–243 (2024).

15. W. Huang, J. Chen, Y. Yao, D. Zheng, X. Ji, L. W. Feng, D. Moore, N. R. Glavin, M. Xie, Y. Chen, R. M. Pankow, A. Surendran, Z. Wang, Y. Xia, L. Bai, J. Rivnay, J. Ping, X. Guo, Y. Cheng, T. J. Marks, A. Facchetti, Vertical organic electrochemical transistors for complementary circuits. *Nature* **613**, 496–502 (2023).

16. C. Cea, Z. Zhao, D. J. Wisniewski, G. D. Spyropoulos, A. Polyravas, J. N. Gelinas, D. Khodagholy, Integrated internal ion-gated organic electrochemical transistors for stand-alone conformable bioelectronics. *Nat. Mater.* **22**, 1227–1235 (2023).

17. J. Lenz, F. Del Giudice, F. R. Geisenhof, F. Winterer, R. T. Weitz, Vertical, electrolyte-gated organic transistors show continuous operation in the MA cm^{-2} regime and artificial synaptic behaviour. *Nat. Nanotechnol.* **14**, 579–585 (2019).

18. M. Forsyth L. Porcarelli X. Wang N. Goujon D. Mecerreyes Innovative electrolytes based on ionic liquids and polymers for next-generation solid-state batteries. *Acc. Chem. Res.* **52**, 686–694 (2019).

19. F. Chen, X. Wang, M. Armand, M. Forsyth, Cationic polymer-in-salt electrolytes for fast metal ion conduction and solid-state battery applications. *Nat. Mater.* **21**, 1175–1182 (2022).

20. E. Stavrinidou, P. Leleux, H. Rajaona, D. Khodagholy, J. Rivnay, M. Lindau, S. Sanaur, G. G. Malliaras, Direct measurement of ion mobility in a conducting polymer. *Adv. Mater.* **25**, 4488–4493 (2013).

21. W. Wang, Z. Li, M. Li, L. Fang, F. Chen, S. Han, L. Lan, J. Chen, Q. Chen, H. Wang, C. Liu, Y. Yang, W. Yue, Z. Xie, High-transconductance, highly elastic, durable and recyclable all-polymer electrochemical transistors with 3D micro-engineered interfaces. *Nanomicro Lett.* **14**, 184 (2022).

22. D. Wang, S. Zhao, R. Yin, L. Li, Z. Lou, G. Shen, Recent advanced applications of ion-gel in ionic-gated transistor. *npj Flex. Electron* **5**, 13 (2021).

23. G. Liu, W. Wen, Z. Zhao, X. Huang, Y. Li, M. Qin, Z. Pan, Y. Guo, Y. Liu, Bionic tactile-gustatory receptor for object identification based on all-polymer electrochemical transistor. *Adv. Mater.* **35**, e2300242 (2023).

24. Y. Lu, C.-Z. Zhao, J.-Q. Huang, Q. Zhang, The timescale identification decoupling complicated kinetic processes in lithium batteries. *Joule* **6**, 1172–1198 (2022).

25. H. M. Fares, J. B. Schlenoff, Diffusion of sites versus polymers in polyelectrolyte complexes and multilayers. *J. Am. Chem. Soc.* **139**, 14656–14667 (2017).

26. P. Batys, Y. Zhang, J. L. Lutkenhaus, M. Sammalkorpi, Hydration and temperature response of water mobility in Poly(diallyldimethylammonium)-Poly(sodium 4-styrenesulfonate) complexes. *Macromolecules.* **51**, 8268–8277 (2018).

27. E. N. Durmaz, S. Sahin, E. Virga, S. de Beer, L. C. P. M. de Smet, W. M. de Vos, Polyelectrolytes as building blocks for next-generation membranes with advanced functionalities. *ACS Appl. Polym. Mater.* **3**, 4347–4374 (2021).

28. M. Khavani, P. Batys, S. M. Lalwani, C. I. Eneh, A. Leino, J. L. Lutkenhaus, M. Sammalkorpi, Effect of ethanol and urea as solvent additives on PSS-PDADMA polyelectrolyte complexation. *Macromolecules.* **55**, 3140–3150 (2022).

29. C. Luo, T. Kuner, R. Kuner, Synaptic plasticity in pathological pain. *Trends Neurosci.* **37**, 343–355 (2014).

30. C. Li, X. Zhang, P. Chen, K. Zhou, J. Yu, G. Wu, D. Xiang, H. Jiang, M. Wang, Q. Liu, Short-term synaptic plasticity in emerging devices for neuromorphic computing. *iScience* **26**, 106315 (2023).

31. S. Y. Wang, X. Z. Chen, X. H. Huang, D. W. Zhang, P. Zhou, Neuromorphic engineering for hardware computational acceleration and biomimetic perception motion integration. *Adv. Intell. Syst.* **2**, 2000124 (2020).

32. G. M. Matrone, E. R. W. van Doremaele, A. Surendran, Z. Laswick, S. Griggs, G. Ye, I. McCulloch, F. Santoro, J. Rivnay, Y. van de Burgt, A modular organic neuromorphic spiking circuit for retina-inspired sensory coding and neurotransmitter-mediated neural pathways. *Nat. Commun.* **15**, 2868 (2024).

33. H. H. Chouhdry, D. H. Lee, A. Bag, N. E. Lee, A flexible artificial chemosensory neuronal synapse based on chemoreceptive ionogel-gated electrochemical transistor. *Nat. Commun.* **14**, 821 (2023).

34. A. Weissbach, L. M. Bongartz, M. Cucchi, H. Tseng, K. Leo, H. Kleemann, Photopatternable solid electrolyte for integrable organic electrochemical transistors: operation and hysteresis. *J. Mater. Chem. C.* **10**, 2656–2662 (2022).

35. J. W. Moon, D. U. Lim, Y. N. Kim, J. H. Kim, J. H. Kim, S. B. Jo, J. H. Cho, High-speed operation of electrochemical logic circuits depending on 3D construction of transistor architectures. *Adv. Funct. Mater.* **33**, 2305440 (2023).

36. S. Y. Jeong, J. W. Moon, S. Lee, Z. Wu, S. H. Park, J. H. Cho, H. Y. Woo, Ion gel-gated quasi-solid-state vertical organic electrochemical transistor and inverter. *Adv. Electron. Mater.* **9**, 2300053 (2023).

37. X. Wu, S. Chen, M. Moser, A. Moudgil, S. Griggs, A. Marks, T. Li, I. McCulloch, W. L. Leong, High performing solid-state organic electrochemical transistors enabled by glycolated polythiophene and ion-gel electrolyte with a wide operation temperature range from −50 to 110 °C. *Adv. Funct. Mater.* **33**, 2209354 (2022).

38. Z. Zhang, X. Yang, P. Li, Y. Wang, X. Zhao, J. Safaei, H. Tian, D. Zhou, B. Li, F. Kang, G. Wang, Biomimetic dendrite-free multivalent metal batteries. *Adv. Mater.* **34**, e2206970 (2022).

39. J. Huang, Y. Zhong, H. Fu, Y. Zhao, S. Li, Y. Xie, H. Zhang, B. Lu, L. Chen, S. Liang, J. Zhou, Interfacial biomacromolecular engineering toward stable ah-level aqueous zinc batteries. *Adv. Mater.*, e2406257 (2024).

40. A. Maradesa, B. Py, J. Huang, Y. Lu, P. Iurilli, A. Mrozinski, H. M. Law, Y. Wang, Z. Wang, J. Li, S. Xu, Q. Meyer, J. Liu, C. Brivio, A. Gavrilyuk, K. Kobayashi, A. Bertei, N. J. Williams, C. Zhao, M. Danzer, M. Zic, P. Wu, V. Yrjänä, S. Pereverzyev, Y. Chen, A. Weber, S. V. Kalinin, J. P. Schmidt, Y. Tsur, B. A. Boukamp, Q. Zhang, M. Gaberšček, R. O'Hayre, F. Ciucci, Advancing electrochemical impedance analysis through innovations in the distribution of relaxation times method. *Joule* **8**, 1958–1981 (2024).

41. Y. Wu, F. Wang, Y. Wu, B. Yan, Q. Li, Advanced ionic actuators with high-performance and high-reproducibility based on free-standing bacterial cellulose-reinforced

poly(diallyldimethylammonium chloride) membranes and PEDOT/PSS electrodes *Cellulose* **30**, 7825–7837 (2023).

42. D. A. Bernards, G. G. Malliaras, Steady-state and transient behavior of organic electrochemical transistors. *Adv. Funct. Mater.* **17**, 3538–3544 (2007).

43. S. T. Keene, J. E. M. Laulainen, R. Pandya, M. Moser, C. Schnedermann, P. A. Midgley, I. M. Culloch, A. Rao, G. G. Malliaras, Hole-limited electrochemical doping in conjugated polymers. *Nat. Mater.* **22**, 1121–1127 2023.
